# The effect of allometric scaling in coral thermal microenvironments

**DOI:** 10.1371/journal.pone.0184214

**Published:** 2017-10-12

**Authors:** Robert H. Ong, Andrew J. C. King, Jaap A. Kaandorp, Benjamin J. Mullins, M. Julian Caley

**Affiliations:** 1 Fluid Dynamics Research Group, Curtin Institute for Computation, Department of Mechanical Engineering, Curtin University, Perth, Australia; 2 Computational Science Section, University of Amsterdam, Amsterdam, The Netherlands; 3 Occupation and Environment, School of Public Health, Curtin University, Perth, Australia; 4 School of Mathematical Sciences, Queensland University of Technology, Brisbane, Queensland, Australia; 5 Australian Research Council Centre of Excellence for Mathematical and Statistical Frontiers, Victoria, Australia; Stockholm University, SWEDEN

## Abstract

A long-standing interest in marine science is in the degree to which environmental conditions of flow and irradiance, combined with optical, thermal and morphological characteristics of individual coral colonies, affects their sensitivity of thermal microenvironments and susceptibility to stress-induced bleaching within and/or among colonies. The physiological processes in Scleractinian corals tend to scale allometrically as a result of physical and geometric constraints on body size and shape. There is a direct relationship between scaling to thermal stress, thus, the relationship between allometric scaling and rates of heating and cooling in coral microenvironments is a subject of great interest. The primary aim of this study was to develop an approximation that predicts coral thermal microenvironments as a function of colony morphology (shape and size), light or irradiance, and flow velocity or regime. To do so, we provided intuitive interpretation of their energy budgets for both massive and branching colonies, and then quantified the heat-size exponent (b*) and allometric constant (*m*) using logarithmic linear regression. The data demonstrated a positive relationship between thermal rates and changes in irradiance, *A*/*V* ratio, and flow, with an interaction where turbulent regime had less influence on overall stress which may serve to ameliorate the effects of temperature rise compared to the laminar regime. These findings indicated that smaller corals have disproportionately higher stress, however they can reach thermal equilibrium quicker. Moreover, excellent agreements between the predicted and simulated microscale temperature values with no significant bias were observed for both the massive and branching colonies, indicating that the numerical approximation should be within the accuracy with which they could be measured. This study may assist in estimating the coral microscale temperature under known conditions of water flow and irradiance, in particular when examining the intra- and inter-colony variability found during periods of bleaching conditions.

## Introduction

Coral biodiversity is essential to provide continuous and well-functioning ecosystems at local and global scales, however, ecological success is strongly coupled to environmental conditions via the thermal sensitivity and acclimation on coral physiological performance. The thermal sensitivity of corals is such that differences in exposures of just ±1°C produce variations in the severity of bleaching and mortality [1, 2]. Despite the significant threat of coral bleaching, bleaching variability and susceptibility to thermal stress within individual and among species still remain unclear, partly due to the complex nature of the interplay between thermal and physiological sensitivity [3, 4], variation in morphology of colonies [5–7], and temporal and spatial conditions experienced by coral microenvironments [8–12]. Importantly, physiology also mediates biotic interactions where energetics needs for thermoregulation change in different environmental contexts [4]. For example, the efficacy of metabolism and behavioural responses depends on the cellular environment (temperature, pH, acid-base balance, etc), which is directly influenced by a shift fluctuation in a rapidly changing environment [4]. Thus, changes in coral body temperature affect biochemical rates and function best within a relatively narrow range. Because the thermal sensitivities of species reaction rates vary, the challenge for corals lies in maintaining the stoichiometry of their complex cellular biochemisty, which will be disrupted by a change in variability of operative coral thermal microenvironments and external temperatures within which corals thermoregulate [4]. Thus, small differences in coral body temperature could have direct implications for our understanding of short-term physiological responses to thermal events in nature.

Considerable spatial and temporal variability exists in bleaching responses to thermal events, both between colonies and within coral species [8, 13–15]. Differential bleaching among colony types may lead to differential mortality [16], which could, in turn, significantly impact the dynamics and structure of coral communities [17–19]. This bleaching variability has been linked to numerous biological factors, including differential susceptibilities of genetic clades of zooxanthellae [20], growth rates [21, 22], thermal tolerances of photoendosymbionts [23], and the influence of photoprotective host pigments [24]. Overall, the role of the diversity and composition of symbiont populations (*Symbiodinium*) associated with thermal stress have received comparatively more attention than that of the role of the coral host, yet it is thermal characteristic of the host colonies’ morphological traits—including colony size, shape, and composition including tissue thickness, permeability, and aragonite density [5, 7, 21, 22, 25, 26]—that is of primary concern. Generally, fast growing and finely branched species of the genera, *Acropora* and *Pocillopora*, are among the most susceptible to thermal stress and suffer higher mortality rates following bleaching [5, 8, 13, 14, 21, 27–38]. In contrast, massive corals such as species of *Porites* and *Favids* are more resistant to thermal stress and more frequently experience partial, rather than whole colony mortality associated with bleaching [5, 21, 22, 39].

One approach to better understanding the relationship between bleaching susceptibility and coral morphology is through observations of thermal exposure in coral microenvironments. The complex physical processes that determine microscale coral temperature is fundamental to our understanding of variation in thermal stress, but how they may respond to stress have been largely overlooked. The coral thermal microenvironment is defined as the temperature of the colony surface and of the boundary layer of water directly next to it, which corals experience directly and may deviate substantially from local sea surface temperatures (SST) [6, 40–42]. Such temperature deviations can be a function of several factors, but predominantly are due to heat flux, radiation and flow dynamics within and surrounding the coral colony [6, 7, 42]. Such dynamics are essential for understanding and monitoring heat-induced stress at reef- and colony-scale. To date, most studies of flow at the colony-scale have used laboratory and computational approaches with idealised geometries and/or flow conditions [6, 7, 41, 43–46]. Therefore, slight modifications in coral surface geometry, as well as thermal and flow properties, could result in variable surface temperatures of individual colonies. Indeed, some experimental studies have demonstrated that thermal stress induced by the coral thermal microenvironment can play an important role in coral bleaching [6, 7, 41, 47]. Recent accumulating evidence also strongly suggests that both flow and light play an important role in coral photosynthesis and respiration, hence affecting primary production in corals [48, 49]. Thus, that some coral colonies tend to bleach more readily than others may be related to relationships between colony morphology and coral thermal microenvironments. Moreover, coral thermal microenvironments are also likely to be important in relation to many other temperature sensitive processes, such as rates of metabolism and growth [50–53].

Body size affects an organism’s structure and functions with most traits scaling disproportionately with size, thus, allometric scaling affects diverse biological variables ranging from physiological to life-history traits [54–58]. The relationship between two variables in an organism is often expressed using the power function *y* = *ax*^*b*^, where *a* is a normalisation constant and *b* is the power exponent. When *b* = 1 the relationship is isometric and when *b* ≠ 1 the relationship is allometric [59]. The widely known and most used scaling exponent that increases in proportion to body mass according to a power of 0.75 [55, 60–62]. But an early proposal of the surface law predicted the scaling exponent between metabolic rate with body surface to be 2/3 [63], so there is still much debate regarding the use of these exponents and why empirical values often diverge from those expected from dimensional analysis [64, 65]. All things being equal, colony scaling is hypothesised to be isometric [66–68]. While there have been experimental tests of this assertion and some support for isometry in corals [69], more cases of allometric scaling have been reported [51, 57, 70, 71]. The morphological factor thought to have the most influence on the thermal rate is the surface area to volume ratio. As expected, colony size and species is closely related to bleaching susceptibility and mortality rates [5, 19, 25, 72, 73]. For instance, much field evidence suggests that small juvenile corals of branching species are more susceptible to thermally induced bleaching than large and mature colonies [5, 74–78]. These studies provide evidence that colony-level differences may indeed affect bleaching susceptibility and suggest that the impact of future thermal anomalies may be more prominent on smaller colonies [26]. However, such size-based patterns of bleaching are not necessarily consistent among sites and species. Instead, bleaching may vary according to the relationship between an individual coral colony and its exposure to coral microenvironments that induce varying degrees of thermal stress related to various environmental factors including location and depth, variations in water flow, and irradiance [6, 10, 21, 41, 79]. Because most coral colonies do not maintain geometric similarity as they grow, we estimated the power values in response to changes in irradiance, *A*/*V* ratio, and flow conditions.

The study of the relationship between bleaching susceptibility and colony morphology has so far been dominated by laboratory and in-situ observations, and predictive modelling studies based on remotely sensed sea conditions, particularly SST. While much has been learned from these studies, they have been inevitably restricted to investigating only a few colonies and few locations due to their relatively high cost and difficulty in obtaining accurate measurements. Another challenge for understanding the causes of coral bleaching over large temporal and spatial scales is the ability to extrapolate from the laboratory scale to spatially extensive patterns of a finite set of key environmental variables such as coral surface area and volume [80, 81], complexity of the spatial flow patterns in the field [82, 83], and variations of irradiance impinging on reefs [9, 11]. The individual relative contributions of these environmental factors (diurnal and tidal variations in flow, water depth, and solar irradiance) to the temporal and spatial pattern of coral thermal dynamics have not been quantified, and the question remains as to how they interact. Numerical modelling techniques, such as computational fluid dynamics (CFD), provide a potential way to address these challenges. CFD is a complete approach that can be used to understand the detailed interactions between heat transfer and fluid dynamics and coral morphologies including varying permeability and thicknesses of the coral tissue and skeleton. Previously, we have demonstrated the efficacy of CFD in estimating the thermal conditions imposed in coral microenvironments under controlled laboratory conditions that included laminar flow and fixed irradiance at a specified overhead angle (90°) [42]. Moreover, our previous study has demonstrated a novel method for combining an accurate ray-tracing technique with CFD to determine coral microscale temperature [84]. However, the overall treatment of the problem described here excludes the approach of the ray-tracing technique. While it is possible to calculate the radiative heat gain and ascertain the total computed irradiance values for a coral surface due to solar position information (azimuth and elevation; or as latitude, longitude and time), these approaches are typically computationally expensive. This study builds on our previous work and is directed towards investigating how thermal microenvironments of corals are affected by the thermal, morphological properties of corals, and how they are influenced by environmental flow and irradiance. The potential thermal effects of allometric scaling in relations to coral shape and size is also examined. The main goal of this work is to predict the range of the heat-size exponent (b*) and allometric constant (*m*) by regressing log-log plots between a set of coupling parameters, namely the dimensionless relations of Nusselt-Reynolds number functions and the generalised thermal scaling due to coral surface area-to-volume (*A*/*V*) ratios. Dimensional analysis of heat transfer modes and water motion within and around corals provides a powerful alternative to traditional allometric methods of investigating the effect of size on thermal rates. Moreover, we present a numerical approximation based on measurable environmental variables in order to produce realistic estimates of microscale temperatures and which we compare to measurements from published experiments.

### Hydro and thermal physics in coral microenvironments

**Fig 1:**
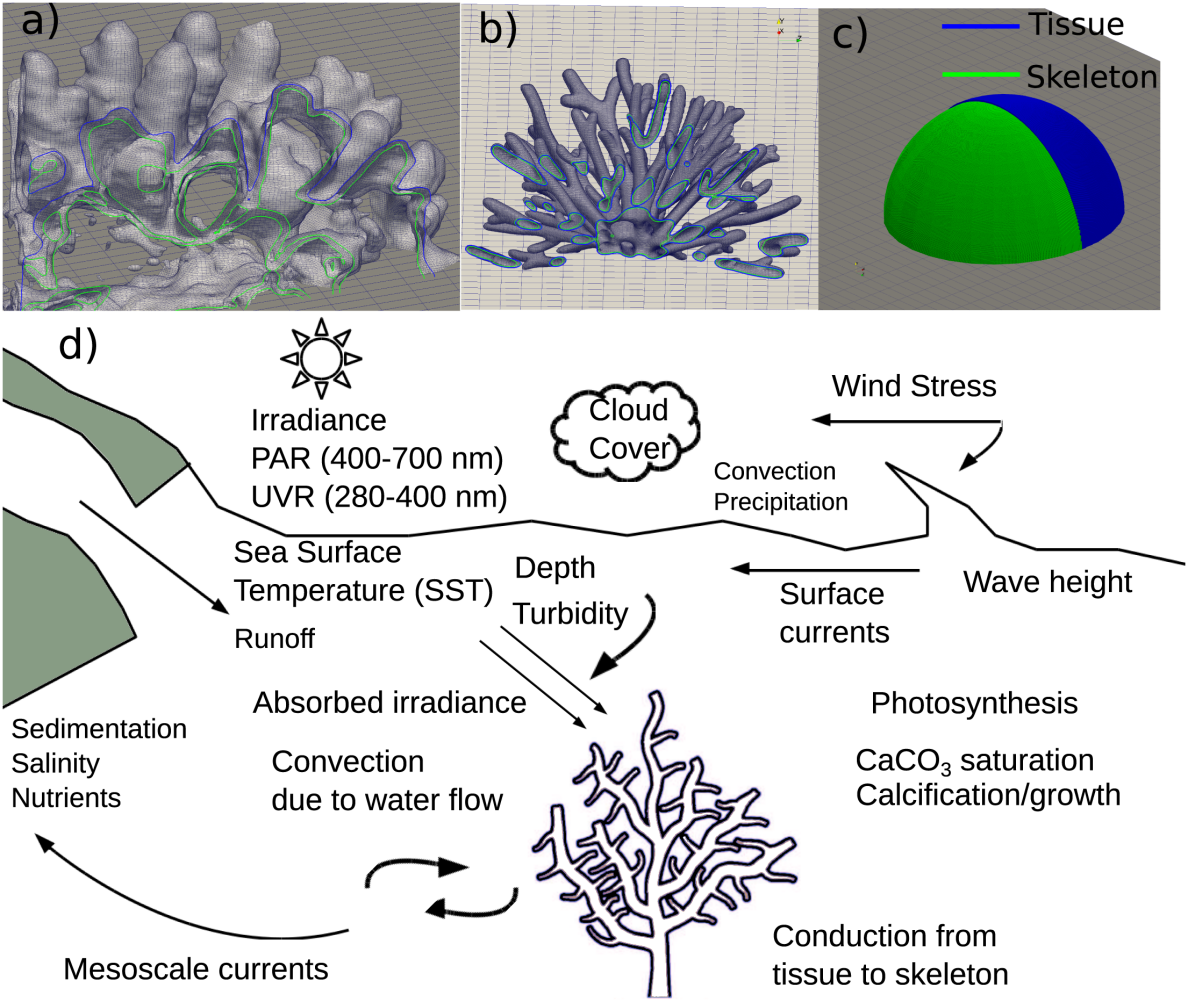
(a), (b), (c): The cross section outlining distribution of living tissue and skeletal matrix in corals, and (d): Schematic diagram summarising key metocean parameters of the coral energy budget in a typical shallow-reef condition.

Environmental conditions of light and water flow, combined with the optical, thermal and morphological characteristics can influence the local thermal environment of corals [40, 85]. The temperature of a coral is a measure of its energy status which is controlled by energy exchange with its surrounding [86]. The models presented here consider two separate thermal and porous flow regions—a thin layer (<10 mm) of living tissue over a thick porous calcium carbonate or aragonite skeleton (Fig 1), but, our models negates the thermal effects due to mass and nutrient transfer during photosynthesis [87].

Our previous mechanistic approach was a simplified representation of a biological system, the polyp architecture was not considered, and only key controlling processes were modelled [42]. The same biophysical method used here was conducted by imposing a simplistic coral structure with smooth surface topographies, and a uniform tissue layer covering the skeleton. Furthermore, the low permeable corals investigated here were based on an idealised condition, such that—in many cases—predated or damaged corals can cause percolation flow. But our model also includes a range of velocity variation we expect to see under real conditions (from impermeable/solid to highly porous). Our previous work has demonstrated the effects of velocity variation and skeletal density on coral thermal microenvironments of the massive and branching corals [42].

The heat transfer within and around corals occurs by three main mechanisms: energy is exchanged with the environment by radiative transfer, heat convection to the water, and heat conduction to the skeleton. As a result, the energy status of a coral is linked to a finite set of environmental variables: solar irradiance, water temperature, and flow. Recent experimental and computational studies provide an analytical framework for the quantitative description of the environmental effects on coral thermal exposure.

#### Conduction from the tissue to the skeleton

The rate of heat conduction from the tissue into the skeleton is the product of the temperature differential between the tissue and the skeleton, the conductivity of aragonite (material of the coral skeleton), and the area over which the contact occurs. Conductive heat transfer can be calculated as:

$$\begin{array}{c} Q_{cond}=k_{s}\;A\;\frac{dT}{ds}∼\frac{k_{s}}{s}\;A\;\left(T_{tissue}-T_{skeleton}\right)=U\;A\;\left(T_{tissue}-T_{skeleton}\right) \end{array}$$ (1)

where $U=\frac{k_{s}}{s}$, and *k*_*s*_ is the thermal conductivity of the aragonite and *s* is the coral’s size. Another variable that affects the rate of conductive heat transfer is the area through which heat is being transferred. For instance, heat transfer through coral tissue to coral skeleton depends on the size of the coral. Corals of larger areas conduct proportionally more heat than smaller ones irrespective of their aragonitic compositions and tissue thicknesses.

#### Convection flux from the sun-exposed tissue to ambient water

Convective heat transport can be assumed to obey Newton’s law of cooling and is proportional to the energy flux or rate of energy loss per second from the surface of the coral with temperature *T*_*tissue*_ into surrounding fluid with temperature *T*_*water*_ and is affected by the pattern and rate of fluid movement around the colony, the amount of surface area in contact with the fluid, and the heat transfer coefficient of water *h*. Convective heat transfer can be calculated as:

$$\begin{array}{c} Q_{conv}=h_{conv}\;A\;\left(T_{tissue}-T_{water}\right) \end{array}$$ (2)

where *h*_*conv*_ is the convective heat transfer coefficient. Theoretically, convective heat transfer is modelled using the Nusselt number, the Prandtl number, the Grashof number and the Rayleigh number. These depend on the Reynolds number which defines the flow regime and the effects of coral morphology on shear stress [51, 88].

#### Absorbed radiative heat flux

For a single radiation wavelength and homogeneous water, the amount of radiant energy absorbed in an interval, Δ*I*, of depth, Δ*z*, is dependent on the amount of irradiance during that interval, which is given by:

$$\begin{array}{c} \Delta I=-\alpha I\Delta z \end{array}$$ (3)

where *α* is coral tissue absorptivity which is equal to the inverse of the optical depth, *I* is the incident of solar irradiance, and *z* is water depth. Eq (3) leads directly to the expression of the radiant energy, *I*(*z*), at depth *z* in the water column in terms of the energy *I*_0_ incident at the surface:

$$\begin{array}{c} I\left(z\right)=I_{0}\text{exp}\left(-\alpha z\right) \end{array}$$ (4)

The heat flux due to absorption of radiation, Δ*Q*, is proportional to Δ*I*, *α*, and the coral surface area (A). Defined as the fraction of incident radiation absorbed by the surface, *α*, accounts for a range of factors affecting the amount of absorbed light, such as the optical properties of both the host tissue (host pigments, tissue thickness) and the zooxanthellae (symbiont density, photosynthetic pigments).

$$\begin{array}{c} \Delta Q=\alpha \Delta IA \end{array}$$ (5)

These modes of heat transfer analysis leads to exponential Newtonian heating or cooling since the amount of thermal energy in the body is directly proportional to its temperature, which in turn determines the rate of heat transfer into, or out of it. Heat transfer is directly proportional to surface area, whereas heat capacity is proportional to volume. The above equation can be rephrased in terms of *q*′′ = *Q*/*A* as:

$$\begin{array}{c} q^{\prime \prime }=\bar{h}\;\left(T_{coral}-T_{fluid}\right) \end{array}$$ (6)

where, $\bar{h}$ is the average heat transfer coefficient over the surface of the body. Without the bar, *h* denotes the local value of the heat transfer coefficient at a point on the surface. Thus, the heat accumulation and dissipation closely depends on coral size and shape, the convective heat transfer coefficient, and the coral’s absorptivity and emissivity.

## Methods

### Modelling framework

#### Allometric scaling in coral thermal microenvironments

Scaling relationships between body size and form and function provide a useful tool for exploring the consequences of differences in morphology among individuals and species [89, 90]. Indeed, our previous proof of concept was developed to predict microscale temperature rises of simplified coral morphology due to a coral’s *A*/*V* ratio under different flow rates and irradiance intensities [42]. The shapes of real corals, however, are unlikely to scale isometrically because coral colonies assume many shapes likely to preclude isometry. To test whether corals scale isometrically or allometrically, two sets of anatomical measurements (*x* and *y*) can be fitted to a power function to determine the scaling exponent. Hence, the general equation for allometric scaling can be written as:

$$\begin{array}{c} y=a\;x^{b} \end{array}$$ (7)

$$\begin{array}{c} \text{log}\;y=\text{log}\;a+b\;\;\text{log}\;x \end{array}$$ (8)

This equation describes a case of functional allometry arising from geometric isometry in relation to body size and thermal scaling in corals, where *a* is the y-intercept and *b* is the slope or scaling exponent with a common predicted value of 0.67 [60, 61]. A number of studies have demonstrated the importance of allometry to mass transfer in predicting metabolic scaling characteristics [51], resource capture [71], and aerobic respiration and photosynthesis [57, 67, 68]. However, to our knowledge no studies have yet applied allometric scaling predictions using heat transfer theory to predict thermal dynamics in coral microenvironments due to morphology, irradiance, and water-flow velocity.

#### Nusselt-reynolds number analysis

The nature of the flow regime around coral colonies is traditionally characterised by the well-known Reynolds number (*Re*), which is the ratio of inertia to viscous forces in the fluid and its order of magnitude serves as an index of physical character (laminar or turbulent) of the flow around submerged objects [91, 92]. The effects of flow on heat transfer at the coral surface were analysed in a dimensionless form. A non-dimensional measure of heat transfer is provided by the Nusselt number (*Nu*)—traditionally defined as the ratio of the heat flux assisted by water motion to the flux that would occur by conduction alone—given by:

$$\begin{array}{c} Nu=\frac{h\;L_{c}}{k_{f}} \end{array}$$ (9)

where, *h* is the convective heat transfer coefficient, *L*_*c*_ is the characteristic length, and *k*_*f*_ is the thermal conductivity of the fluid. But, the context of *Nu* here is not defined as the ratio of convection to conduction, rather a non-dimensional heat transfer coefficient. This heat transfer coefficient is governed by operating parameters such as size and shape of the coral colony, mass flux, and pressure, as well as physical properties of the fluid such as its density, specific heat, viscosity, and thermal conductivity. The characteristic length of coral colonies is the dimension that defines the length scale of corals normal to the substrate along the direction of boundary layer development because these gradients are usually the largest, generating most of the heat, mass, and momentum transport. Heat transfer coefficients are related to tissue thicknesses, shapes, and sizes of coral colonies by an inverse power function because larger objects will have absolutely thicker boundary layers for the same flow speed relative to small objects. The Nusselt number can be calculated locally as a function of the position downstream (*x*) over a coral colony (*Nu*_*x*_), but doing so would require calculations with surface warming of the local heat fluxes within patches of the colony. Hence, the *Nu*_*x*_ is integrated over the whole, branching, or massive colonies to determine $\bar{Nu}$, the average Nusselt number. The whole or module assemblage is the combination of branching and massive colonies datasets.

The effects of forced convection on heat transfer is characterised using plots of *Nu* (ordinate)—*Re* (abscissa). This relationship can be expressed as a power law:

$$\begin{array}{c} Nu=a\;Re^{b} \end{array}$$ (10)

where *a* and *b* are heat proportionality coefficients and heat exponent values, respectively, and in logarithmic form is:

$$\begin{array}{c} \text{log}\;Nu=\text{log}\;a+b\;\text{log}\;Re \end{array}$$ (11)

The curvature of the *Nu* and *Re* relationships can then be used in this linear format to yield values of a* and b* which are the global heat coefficient and exponent terms, respectively. These values are directly affected by a coral’s shape and the flow regime around it, and thus will vary among colonies [51]. The heat exponent, *b*, is usually important in representing the degree to which flow augments convective heat transfer. Heat transfer is determined by the physical process whereas *b* is a coefficient, and its numerical value indicates whether transport is occurring through a laminar or turbulent boundary layer around the exchange surface. Hence, it follows that *h*_*conv*_ depends on viscosity, thermal conductivity of the fluid and coral, and also nonlinearly on the temperature difference, i.e. *h*_*conv*_ ∼ Δ*T*^*b*^, where *b* depends on flow conditions. In particular, a value of *b* = 0.5 is often used under laminar flow, while *b* > 0.6-0.8 characterises a turbulent flow [91]. Additionally, replacing *Nu* by Sherwood number in Eq (10) (*Sh*—defined as the ratio of oxygen mass flux assisted by water motion to that by diffusion alone, $Sh=\frac{h_{m}\;L_{c}}{D}$) provides the analogous power law relationship for mass transfer. The effects of forced convection on metabolic rates can be characterised using plots of Sherwood and Reynolds (*Sh* − *Re*) numbers.

### Computational fluid dynamics (CFD)

The CFD study was conducted using the OpenFOAM (Computational Fluid Dynamics Software) [93]. A CFD study consists of three main processes—pre-processing, solving, and post-processing. During pre-processing, a geometry is created, which then is divided into discrete cells often referred to the mesh. The physical conditions are defined by specifying material properties and operating conditions, then an iterative method is used to solve for a wide spectrum of phenomena involving fluid flow. Post-processing is required to evaluate and extract solutions. The cornerstone of CFD is the fundamental governing equations of fluid dynamics—the continuity, momentum and energy equations. These equations are defined by partial differential equations obtained directly from the physical principles over the computational mesh. These conservation equations are often called the Navier-Stokes equations. The governing equations below—written in Cartesian coordinates—apply to a fully-developed steady laminar incompressible flow in porous media. The additional transport phenomenon of mass diffusion is not included because the scope of this study is limited to a homogeneous, non-dissolved chemical species reaction. If diffusion were to be included, there would be additional continuity equations—the species continuity equations involving mass transport of chemical species *i* due to a concentration gradient of the species. A detailed CFD modelling and their governing equations used in this study is presented in S1 Text.

### Model assembly and configurations

The characteristic length of three-dimensional branching and massive coral models is varied ranging from millimeters to meter in units scale in order to assert a range of surface area-to-volume (*A*/*V*) ratios. Physical models of various coral species were used to determine the range of heat exponent values and the degree to which water motion and morphology might affect heat transfer, and ultimately, the thermal microenvironments experienced by corals. The model of coral species investigated in this paper and extended description of their characteristic dimensions are given in S2 Text.

To examine the effect of allometry on thermal scaling in coral microenvironments, each model morphology was scaled in size in order to explore a range of *A*/*V* ratios. These profound changes in size are admittedly simplistic, without focusing on growth and ignoring a variety of other important ecological processes (e.g., recruitment, competitive interactions, disturbances, etc). Nonetheless, this facilitates data synthesis and allows us to predict thermal responses of various morphologies. Each coral model was divided into a tissue and a skeleton region with a different respective permeability [42]. These variations were constructed in order to evaluate the effect of convective heat transfer on to the overall microscale coral surface temperature. In many cases, borers and coral grazers can cause openings which allow some degree of percolation and increased skeletal porosity. In order to capture such effects, we modelled a very small percolation flow along the tissue layer (∼1-2%) and a moderate flow through the skeletal matrix (∼7-10%) [42]. In this study, the thermal conductivity values of tissue (mesoglea and silicone) and skeleton (aragonite) were set to 0.22 and 2.1 W m^−1^K^−1^, respectively [94, 95]. The corals’ absorptivity values (*α*) were all set to 0.4. The conceptual representation of this study was designed to simulate coral thermal microenvironment conditions within a flow chamber (S2 Fig). We applied different size chambers based on their flow regimes: 15 cm h (height) × 10 cm w (width) × 25 cm l (length) for laminar simulation, and 15 m h × 10 m w × 25 m l for turbulent simulation. In our simulations, a steady-state unidirectional stagnant flow was maintained at 0.01 m s^−1^, with temperature set at 26°C (293 K). Heat was provided by direct sunlight, where corals were exposed to a maximum irradiance of ≈650 W m^−2^.

### Validations of the CFD results and numerical approximations

Coral surface warming was estimated as the difference between coral surface temperature and ambient water temperature after a few minutes of exposure to direct solar irradiance. Previously, we established the potential of CFD for predicting the thermal stress imposed on branching and massive coral microenvironments due to irradiance and pigmentation under both laminar and turbulent flows [42, 96]. Here, we validated our proposed numerical analysis of coral surface warming against published experimental observations in both laminar [6] and turbulent [41] flows, relevant heat fluxes, absorptivity levels (where *α* = 0.1 (or *F*_0_ = 100) denotes near white and *α* = 0.5 (or *F*_0_ = 500) denotes dark brown). We then assessed the linearity between the previously simulated CFD results and the predicted coral surface warming using linear regression fitted with ±10% and ±20% error margins.

The overall framework of numerical simulations performed in this study is described in S3 Text. The numerical predictions of allometric thermal scaling in corals can be readily determined by regressing log-log plots of $\left(\frac{\Delta T}{Re^{\bar{b^{*}}}}\right)$ (ordinate)—*A*/*V* (abscissa) that yield information about the degree to which water flow (*Re*) and coral surface area-to-volume (*A*/*V*) ratio affect heat exponent (b*) as shown in Eq (8). The linear regression of allometric curve fits is the most appropriate for studies utilising our approach, where the spatial extent and *Re* dependence of flow separation phenomena were not characterised. In order to quantitatively assess the validity of the flow scenario used in this study, we tested the effect of flow variation used in laminar and turbulent flows ranging from 1 cm s^−1^ to 10 cm s^−1^ under a constant irradiance of 650 W m^−2^ on the *Nu*−*Re* exponent values and the generalised allometric constant values.

## Results

### Flow and thermal patterns within and around corals

**Fig 2:**
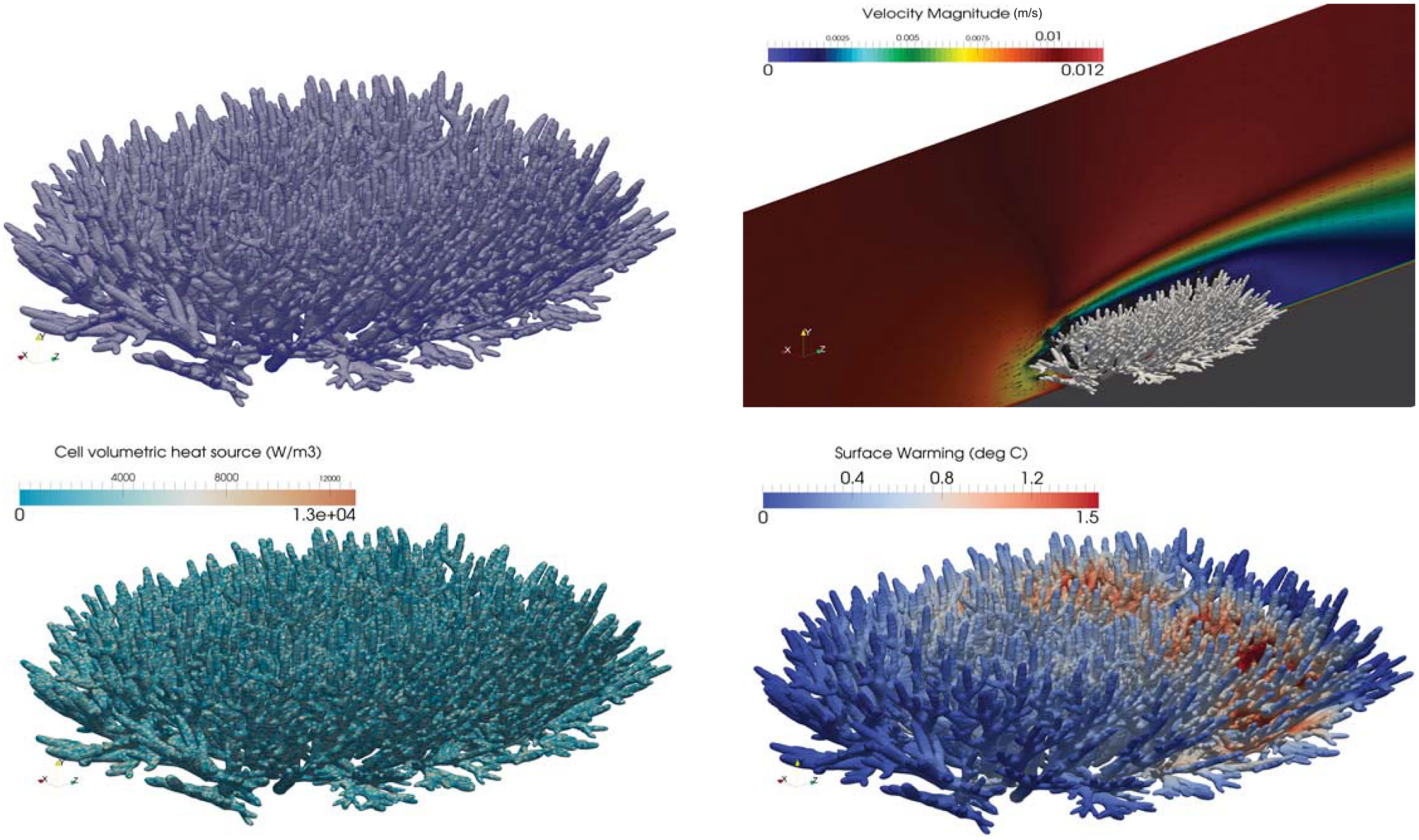
Flow and thermal patterns within and around *Acropora millepora*.

**Fig 3:**
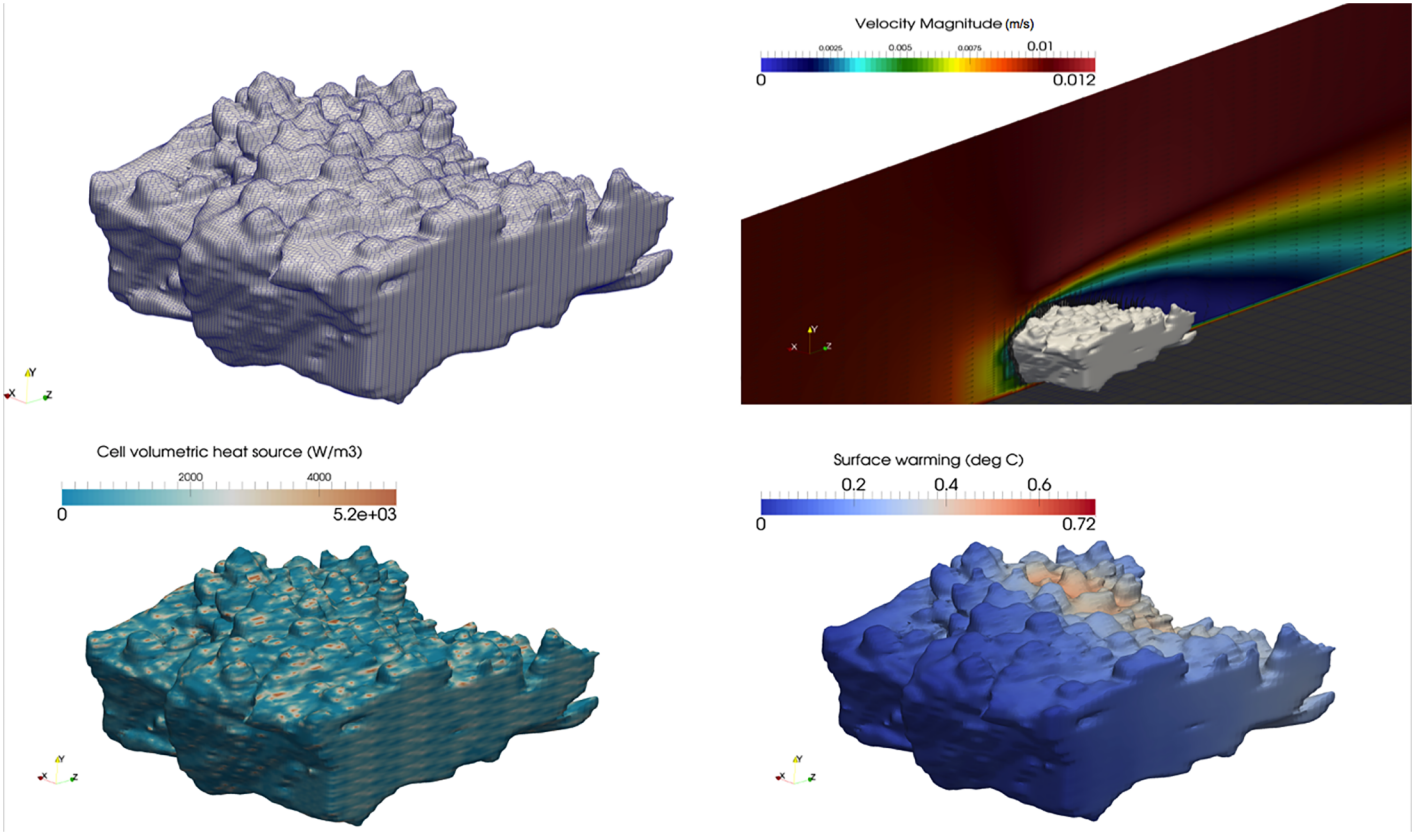
Flow and thermal patterns within and around *Acropora digitifera*.

We provided visualisations of thermal and flow patterns for two branching corals (Fig 2a: *Acropora millepora* and Fig 3a: *Acropora digitifera*). The results of the heat source field calculation are presented in Figs 2c and 3c. The volumetric heat source term is dependent on the volume of the cell, the intersection of the cell and the coral geometry, and the irradiance value itself. Figs 2b and 3b show the flow field in and around the coral with cross-sectional planes highlighting the flow through porous corals. As expected, the flow velocity in the vicinity of and through the coral is reduced significantly, which provides the cooling effects to the coral. Figs 2d and 3d display temperature on the surface of the corals. For the simulated conditions, predicted maximum surface warming (defined as a deviation from the ambient water temperature, 26°C or 299 K) of *A. millepora* and *A. digitifera* were ∼ 1.5°C and 0.7°C, respectively. At the ‘leading edge’ of the coral, there was a significant cooling effect on the coral surface due to the incoming flow. Conversely, higher temperatures were observed at the trailing edge, even though the volumetric heat levels were lower.

### Heat exponents from *Nu* − *Re* plots

**Fig 4:**
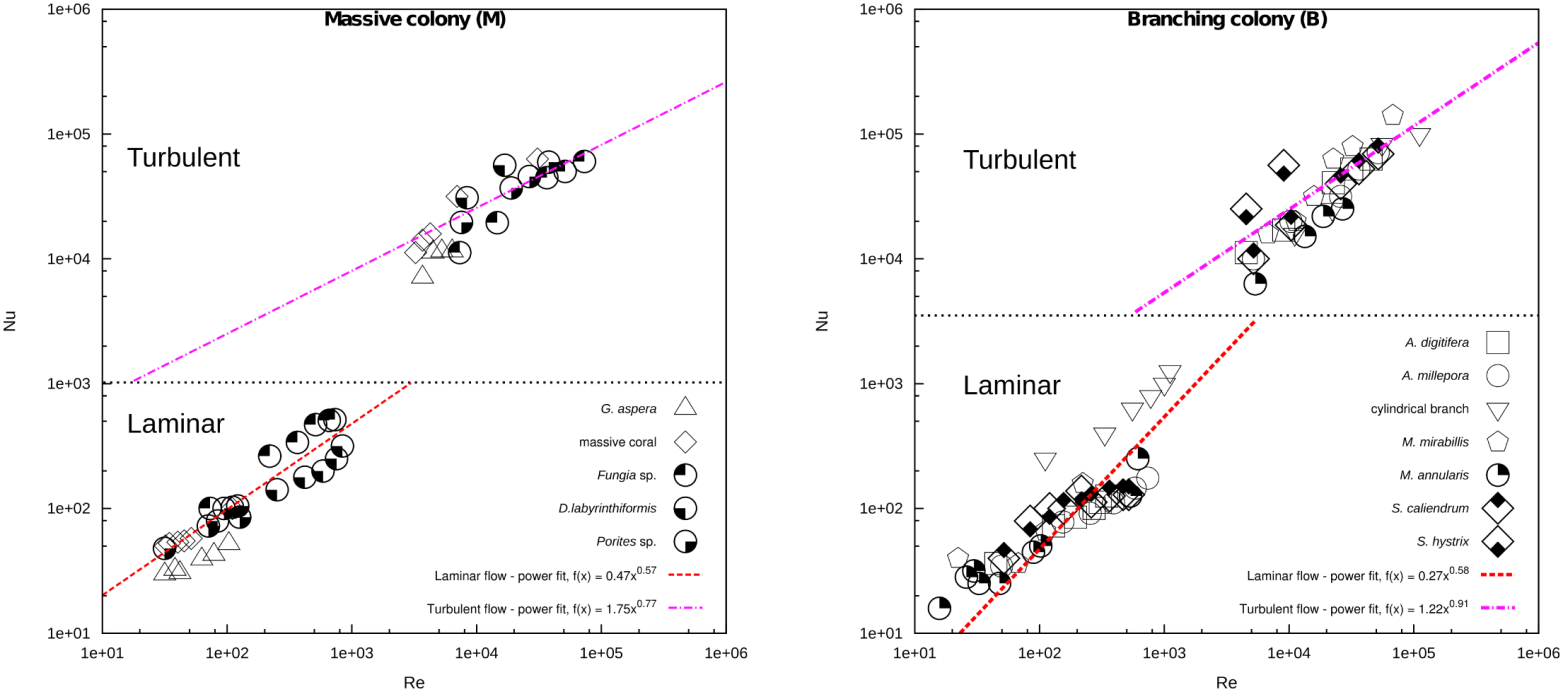
*Nu* − *Re* plots of only the massive colony (M) and branching colony (B) in both laminar and turbulent regimes.

The exact formulation of the dimensionless measure of heat transfer provided by the *Nu* − *Re* number functions (i.e. the values of the coefficients *a* and *b* in Eq (10)) depends on the specific shape of the coral from simple geometry to more complex ones. The resulting *Nu* − *Re* was correlated in log-log space which yield both the heat coefficient and exponent values in laminar and turbulent flows of the massive and branching colonies (Fig 4), and the whole colony (S3 Fig). The effect of flow velocity variations (0.01, 0.002, and 0.001 m s^−1^) on the Nusselt number and their influence on *Nu* − *Re* exponent values are given in S4 Text.

Note that the heat exponents in laminar flow have lower values compared with the turbulent flow. Increased colony size leads to increased *Re* for a given flow speed, which will shift the heat transfer processes further up the *Nu*/*Re* curves. Thus, colony shape and size and the flow regime have a direct impact on the value of the heat exponents obtained. For each shape category under combined laminar and turbulent regimes yielded $\bar{Nu}=0.31\times Re^{1.15}$ for branching colonies and $\bar{Nu}=0.54\times Re^{1.12}$ for massive colonies. The $\bar{Nu}$ indicates the behaviour of heat transfer relative to diffusion and boundary layer thickness. The $\bar{Nu}-Re$ data were fitted to an expression of the form: $\bar{Nu}=aRe^{b}$, where the slope, *b*, and the intercept, *a*, were obtained by regressing the least-squares linear function from the log-log plots, are given in Table 1. An analogous pattern exists in the comparison of slopes derived from various flow speed, implying that the results in this study may be applied to typical flow boundaries that occur within the individual sites on a single coral colony.

**Table 1:** Global heat proportionality coefficient (${\bar{a}}^{*}$) and exponent (${\bar{b}}^{*}$) values from $\bar{Nu}-Re$ plots at flow ranged from 1-10 cm s^−1^.

Flow (m s^−1^)	Constants	Whole assemblage		Branching		Massive	
		Laminar	Turbulent	Laminar	Turbulent	Laminar	Turbulent
U: 0.01	${\bar{a}}^{*}$	0.39	1.49	0.27	1.22	0.47	1.75
	${\bar{b}}^{*}$	0.58	0.85	0.58	0.91	0.57	0.77
U: 0.002	${\bar{a}}^{*}$	–	–	0.81	–	0.61	–
	${\bar{b}}^{*}$			0.56		0.57	
U: 0.001	${\bar{a}}^{*}$	–	–	0.39	6.76	0.78	5.81
	${\bar{b}}^{*}$			0.61	0.85	0.57	0.71

### Allometric thermal scaling exponents

**Fig 5:**
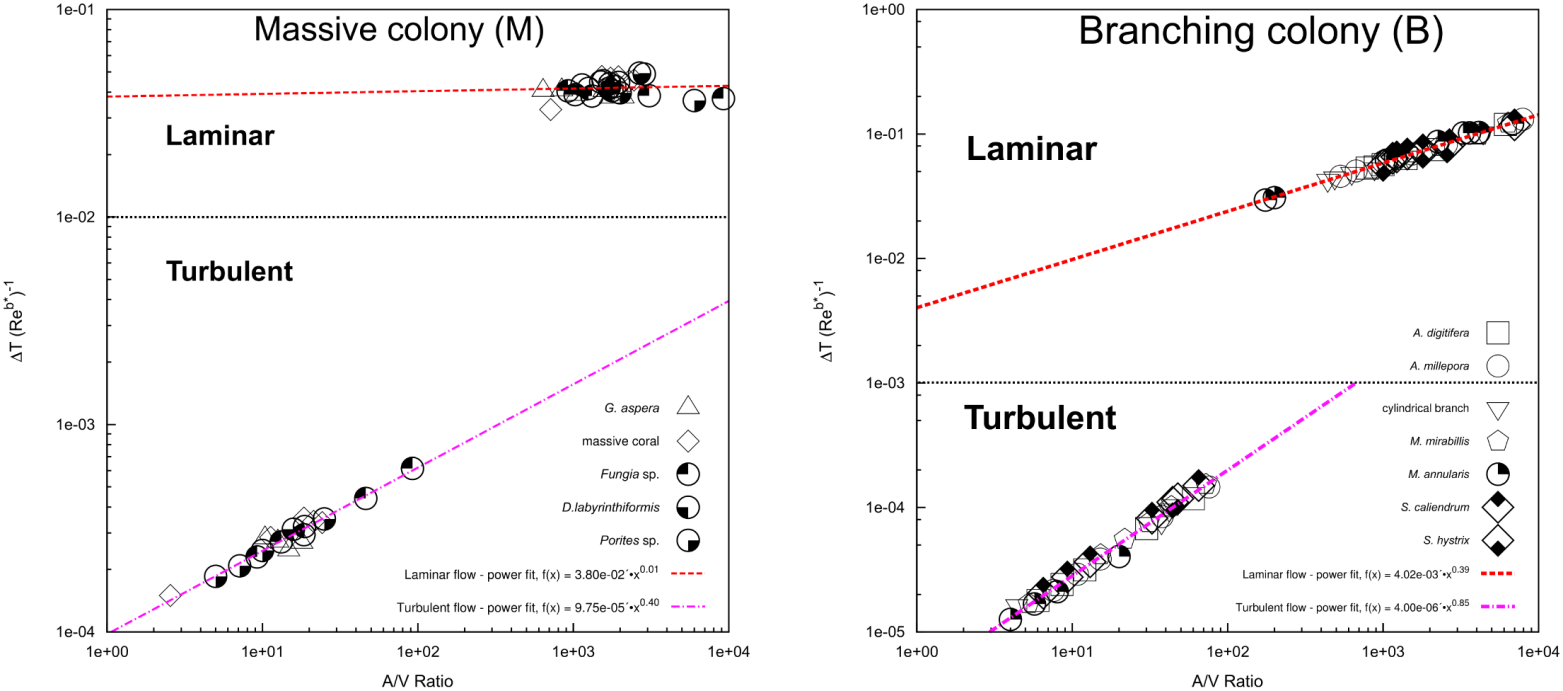
Generalised allometric model constants $\left(\frac{\Delta T}{Re^{\bar{b^{*}}}}-A/V\right)$ of massive (M) and branching colonies (B) at a constant flow velocity of 0.01 m s^−1^.

We established a numerical approximation (Eq (12)) of coral surface warming for any combination of colony shape and size, water-flow velocity, and irradiance, which can be represented by:

$$\begin{array}{c} \Delta \;T=C\;\;{\left(\frac{A}{V}\right)}^{m}\;\;Re^{\bar{b^{*}}}\;\;\alpha \;\left(\frac{I}{I_{0}}\right) \end{array}$$ (12)

where *α* is the coral light-absorptivity or pigmentation coefficient. The term $\left(\frac{I}{I_{0}}\right)$ is normalised irradiance intensity and the initial irradiance, *I*_0_, was approximately 650 W m^−2^. The heat scaling exponents *b* were then fitted to allometric thermal scaling $\left(\frac{\Delta T}{Re^{\bar{b^{*}}}}\right)$ against the *A*/*V* ratio log-log plots of branching and massive colonies (Fig 5) for varying flow velocities ranging from 1-10 cm s^−1^ in order to obtain values for the allometric exponent (*m*) and constant (*C*) values (Table 2). The generalised allometric model constants of the whole assemblage are shown in S6 Fig. *C* is an allometric constant and has units of [°*Cm*^*m*^]. The effect of flow velocity variations (0.01 and 0.001 m s^−1^) on the allometric thermal scaling exponent values is described in S4 Text. The derived heat exponents and allometric constants for various colony shapes at constant flow of 10 cm s^−1^ is given in S7 Table, with exponents similar to published mass transfer exponents values [51].

**Table 2:** Global allometric model constants obtained from log-log regressions of the generalised allometric thermal scaling plots $\left(\frac{\Delta T}{Re^{\bar{b^{*}}}}\right)$ against the surface area to volume (*A*/*V*) ratios for flow velocity ranged from 1-10 cm s^−1^.

Flow (m s^−1^)	Constants	Whole assemblage		Branching		Massive	
		Laminar	Turbulent	Laminar	Turbulent	Laminar	Turbulent
U: 0.01	*C*	7.94 × 10^−3^	1.58 × 10^−5^	3.95 × 10^−3^	4.85 × 10^−6^	2.46 × 10^−2^	9.82 × 10^−5^
	*m*	0.27	0.65	0.39	0.80	0.06	0.40
U: 0.002	*C*	–	–	7.47 × 10^−3^	–	6.00 × 10^−2^	–
	*m*			0.39		-0.03	
U: 0.001	*C*	–	–	8.19 × 10^−3^	1.66 × 10^−5^	7.42 × 10^−2^	1.71 × 10^−4^
	*m*			0.39	0.79	-0.05	0.40

### Predicted coral thermal microenvironments

**Fig 6:**
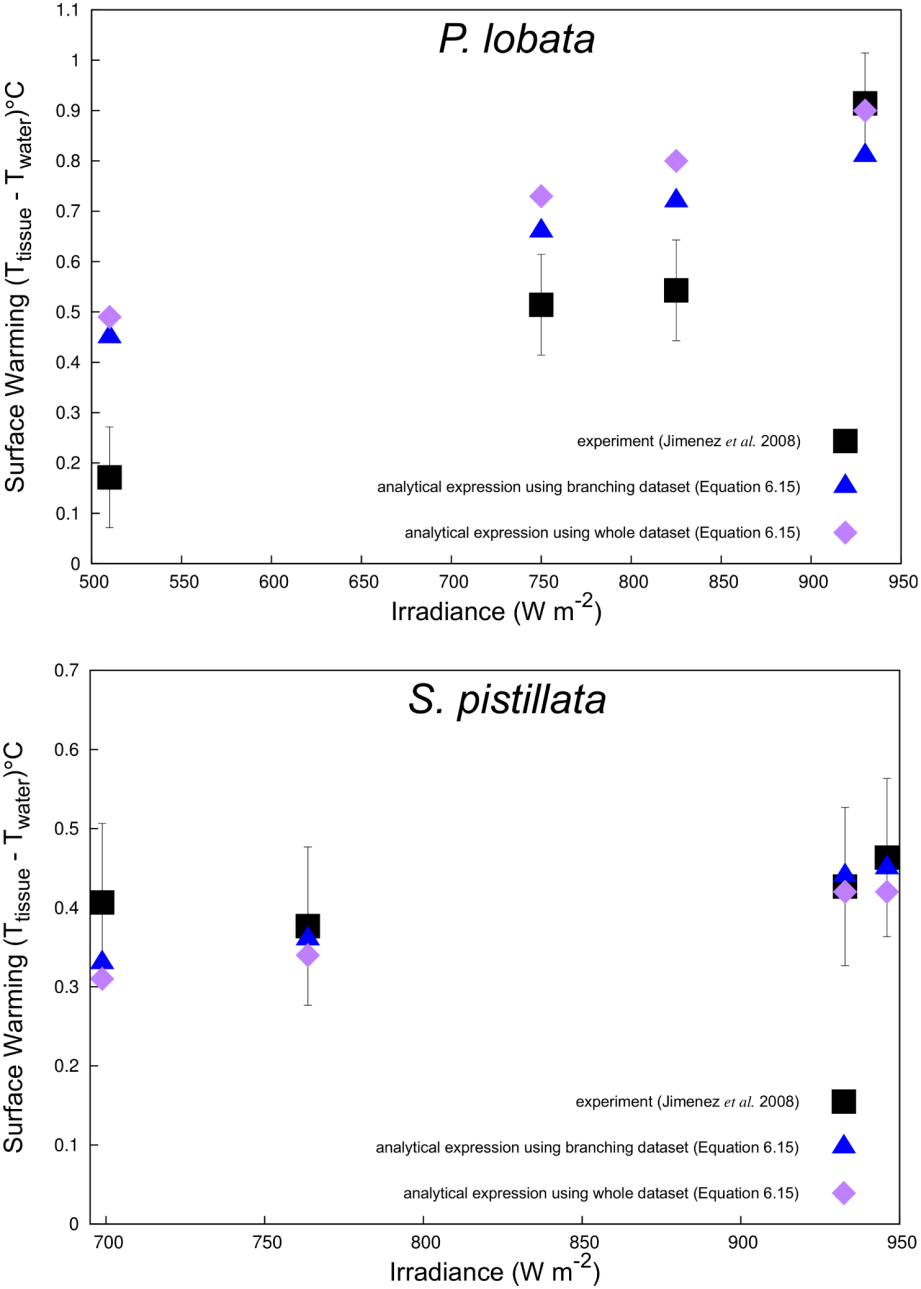
A comparison of coral surface warming between experimental observation [6] and the numerical approximation of laminar flow (Eq (12)) for the massive coral (*P. lobata*∼35 mm) and a section of cylindrical branch (*S. pistillata*∼6 mm).

**Fig 7:**
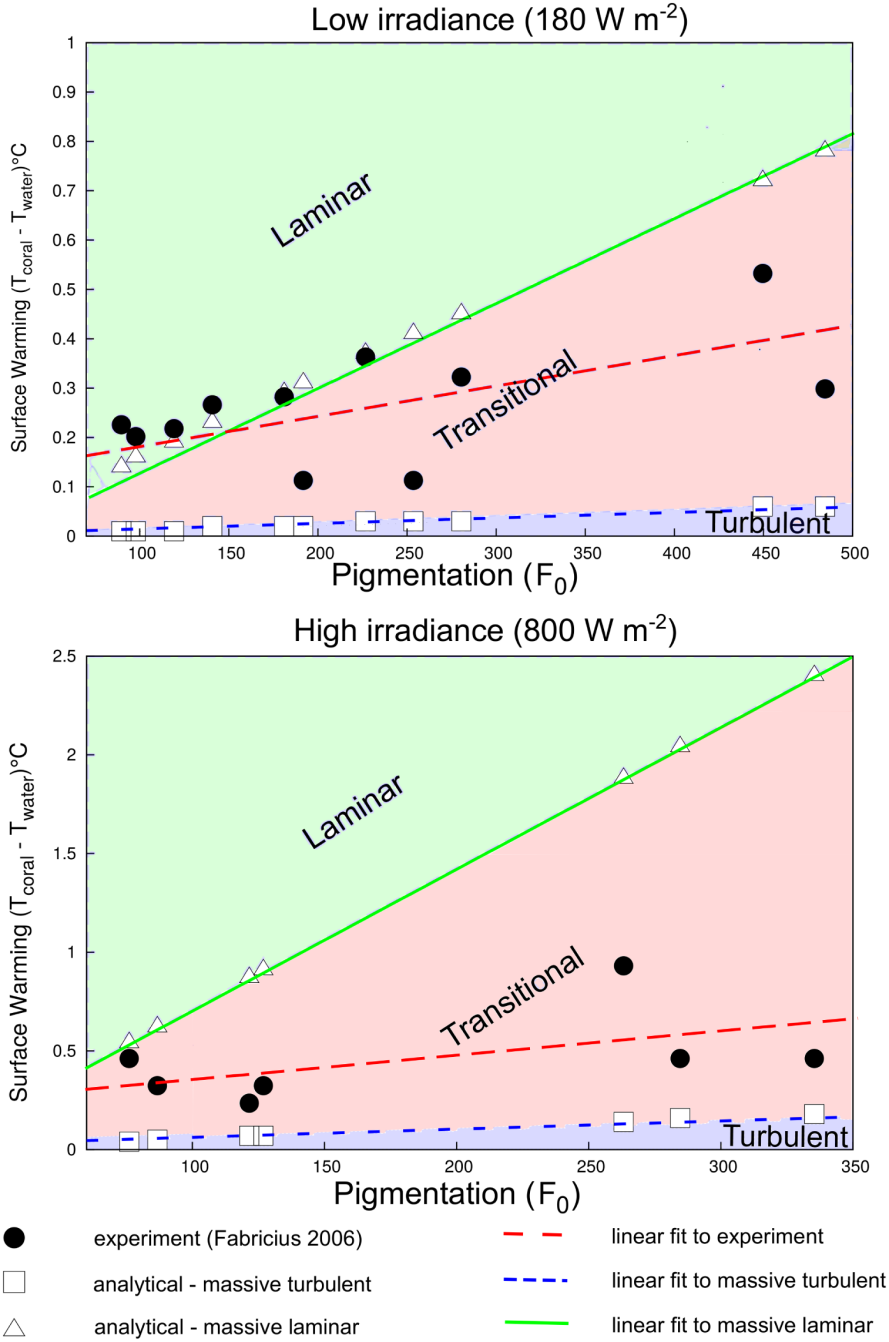
A comparison of coral surface warming due to low and high irradiance levels, and absorptivity coefficients between experimental observation [41] and the numerical model of both laminar and turbulent flows (Eq (12)) for the massive coral (*Favia matthaii*∼90 mm).

**Fig 8:**
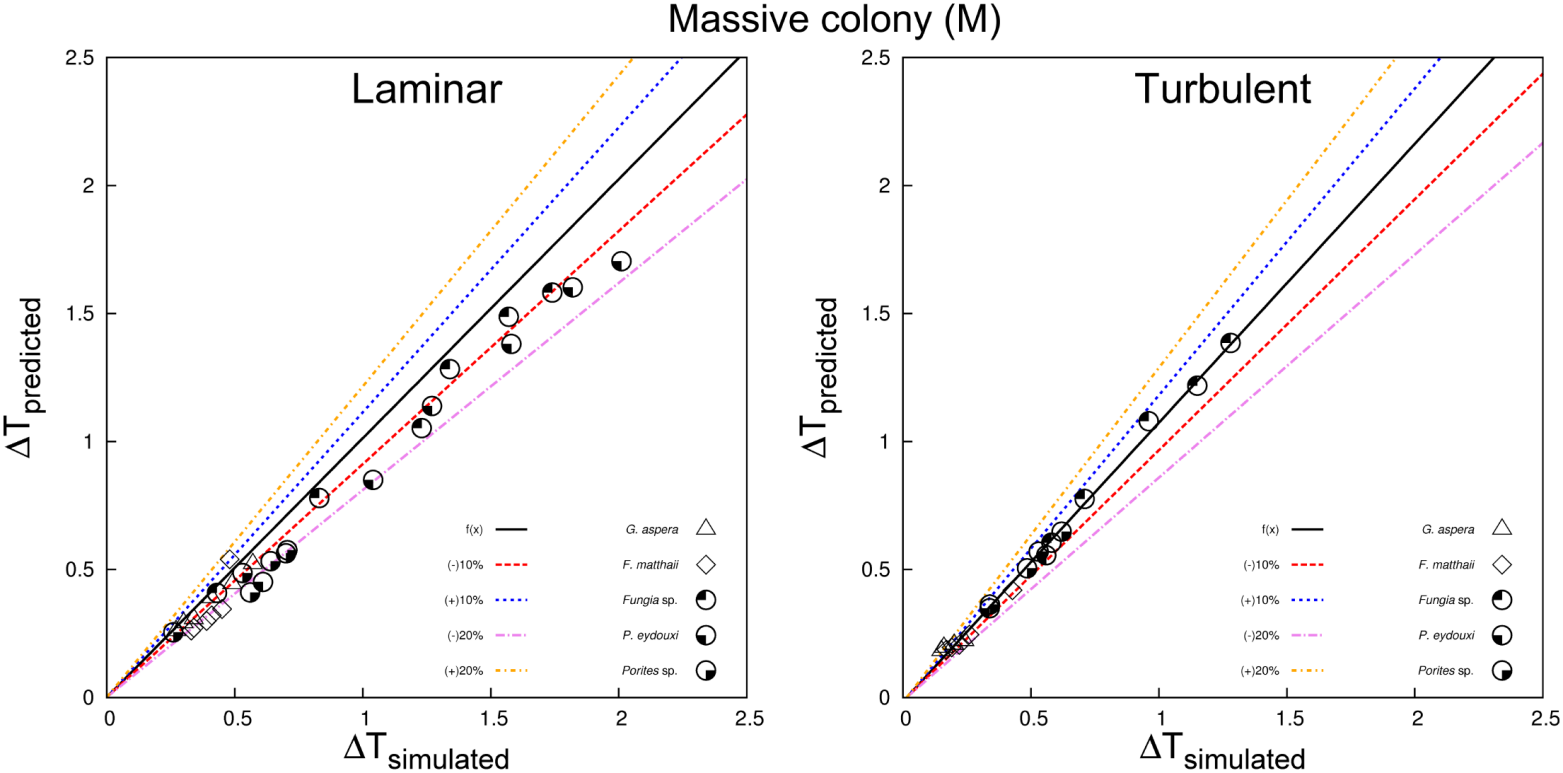
Relationships between Δ*T* for simulated against numerical predictions for the massive colonies (M) in both laminar and turbulent regimes.

**Fig 9:**
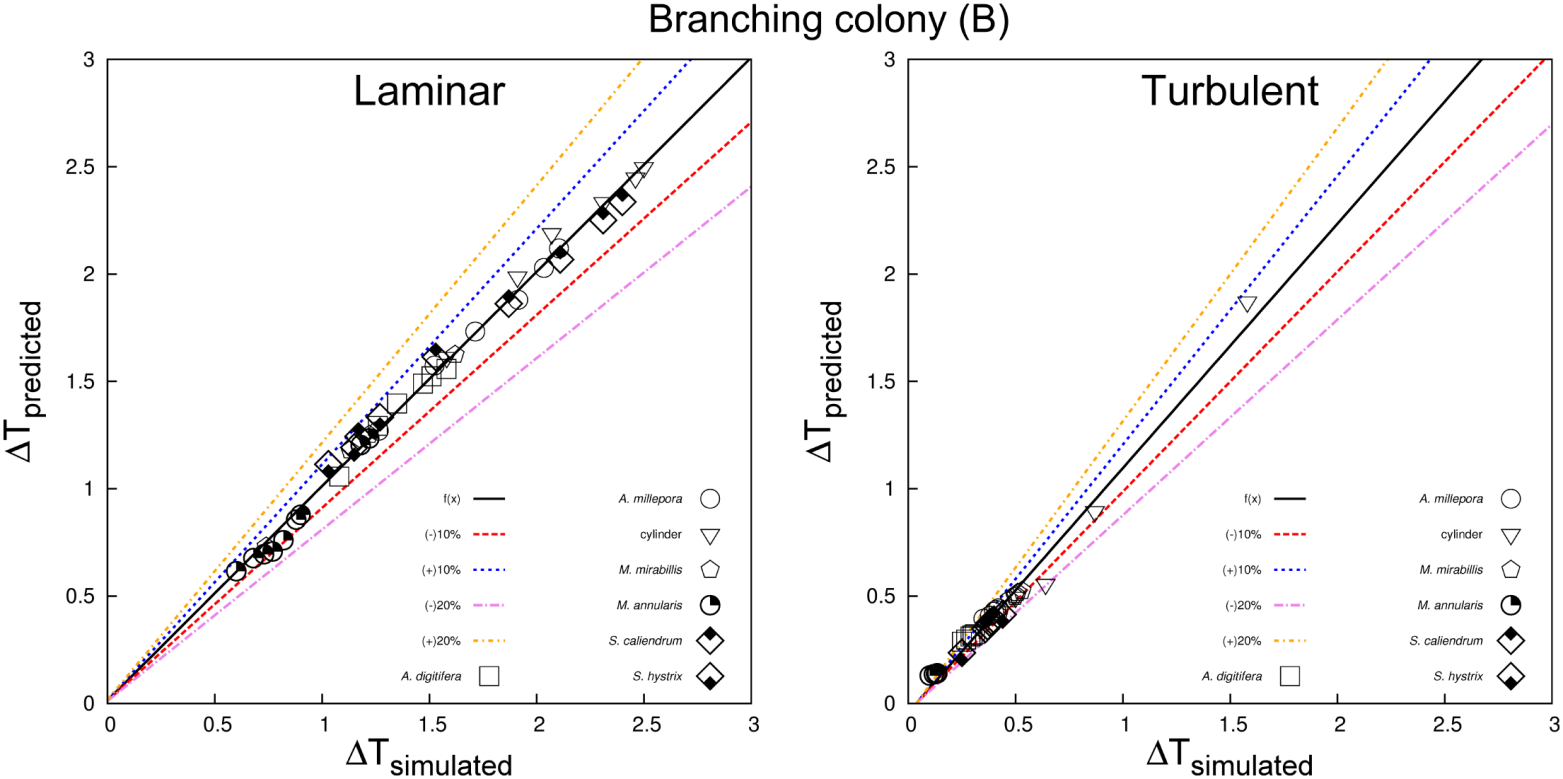
Relationships between Δ*T* for simulated against numerical prediction for the branching colonies (B) in both laminar and turbulent regimes.

Our estimated coral surface warming under laminar flow agreed very well with experimental observations (Fig 6). The numerical prediction also produced a good linear fit between the absorptivity coefficient (*α* or *F*_0_) and coral surface warming, which was also consistent with heat transfer theory. We also classified the laminar, transitional, and turbulent regions based on the derived values of predicted surface warming given in Eq (12) (Fig 7). The scatterplots of the branching and massive colonies illustrate excellent agreements between predicted and simulated values with no significant bias for either the massive (Fig 8) and branching (Fig 9) colony datasets. But, some bias was found in the scatterplot for a whole assemblage S9 Fig, thereby emphasizing the role of coral shape in the overall surface temperature distributions at microscale.

## Discussion

Understanding the complex physical processes that underlie the microscale coral temperatures is essential to our understanding of variation in thermal stress, and how coral may respond to future stress events. Much attention has been paid to investigating bleaching responses due to diversity and composition of the host’s symbiont populations [97–99], however, the role of colony morphology in relation to thermal stress is important in predicting bleaching susceptibility. Studies have demonstrated that the surface temperature of shallow water corals can in fact exceed that of the surrounding water, mainly due to the absorption of solar irradiance [6, 41]. The thermal stress associated with high temperatures, induced by a combination of multiple environmental factors is likely to trigger and determine the severity of bleaching. The relative importance of various physical factors in controlling microscale temperatures depends on how strongly combined environmental factors, such as weather conditions, current flows, etc, affect the energy budget of the coral tissue. Indeed, the effects of these environmental factors on coral temperature are evident, and studies have shown that ocean currents, tides, and wind [10, 50, 100–102], and different growth forms [5, 21, 22], and irradiance levels [9, 11, 76] have been related to whether or not corals bleach. Because small increases in temperature above bleaching thresholds can trigger bleaching [103–106], greater understanding of coral thermal microenvironments should facilitate greater understanding of the causes of coral bleaching.

The physical mechanisms modelled here are deterministic, and can be used to predict variations in microscale coral temperatures. This study provides a numerical approximation of microscale temperature within branching and massive morphologies exposed to solar irradiance and low water flow, using well known concepts from dimensional analysis of heat transfer theory. We used a heat budget model of a coral to describe surface warming in steady-state laminar and turbulent flows, in terms of the relevant heat transfer mechanisms (radiation, convection, and conduction), as well as the coral’s geometrical parameters (surface area to volume ratio). The differential variation with size of area- and volume-dependent phenotypic traits is a primary cause of allometric constraints ranging from physiological to life-history traits [55]. Any deviations of allometric from isometric have been attributed to changes in morphological factors such as changes in the *A*/*V* ratio and the volume fraction of inactive materials as the body size increases [65]. Morphological traits exhibit diverse biological scaling relationships with body size among species and populations, such that the rate at which trait size changes with overall body size often can be nonlinear [107]. Coral colonies of the same species often exhibit very different thermal exposure of similar temporal and spatial scales, which may vary based on morphological characteristics including size and shape [5, 19, 21, 25, 108], geographical location [79] and depth of water column [21]. Considering the acute thermal sensitivity of corals, variations in their thermal environment have been explored previously [6, 41, 42, 47]. Unlike many of these earlier studies, we have focussed on coral thermal microenvironments to help understand heat transfer between coral tissue and seawater and coral convective heat transfer [6, 7, 42]. Differences in coral size and shape can be sufficient to alter heat transfer though the allometric scaling of thermal rates. Therefore, our findings along with other observations suggest that colony size more than its shape, largely accounts for differences in thermal exposure among species [5, 76–78].

The numerically derived relationship between *Nu* and *Re* given in S7 Table is one of the several forms that can be used to describe the effects of flow on heat transfer. Overall, laminar flow results in smaller heat exponents (*b*) compared to turbulent flow. Coral shape and flow regime directly effect the value of heat exponents and allometric constants, thus shape-related differences of thermal exposure may describe some of the observed differences in bleaching susceptibility. The range of heat transfer exponents characterising the effect of flow on surface warming is consistent with expectations from heat transfer principles for basic geometrical shapes [109–111], but further exploration of complex surface topography and geometry is needed to provide a more complete picture of these processed. On theoretical grounds, the heat exponents for flow past an immersed object such as a flat plate, sphere, or cylinder are approximately 0.5 and >0.6-0.8 in laminar and turbulent regimes, respectively [50, 91, 112]. Increases in colony size and/or flow speed increased the rate of convective heat transfer rate between the coral microenvironment and the immediate surroundings and was consistent with Newton’s law of cooling. Additionally, as mentioned above, the theoretical analogy between heat and mass transfer implies that both thermal and metabolic rates are likely to be interchangeable due to a change in flow velocity [6, 7, 109]. Theoretical guidelines from classical heat transfer, together with predictions from our model, can be used to estimate coral surface warming at higher and lower flow rates. Note that *Re* and *Nu* increase with characteristic length (*L*_*c*_) and water flow velocity (*U*), but that increases in heat loss rate (*Nu*) begin to level off at higher Re. Dimensionless analysis of heat transfer confirmed that convective heat transfer at the surface of corals are consistent with predictions from engineering theory for simplified geometrical objects.

The corals investigated here are typically classified as either massive or branching types. In all simulations, the larger massive colonies experienced greater surface warming than did branching corals. This study demonstrates that the *A*/*V* ratio declines with increasing colony size, and vice versa. Thus, the bigger relative surface areas of the small branching colonies (high *A*/*V* ratios) are unlikely to significantly affect the present result of ∼0.4-0.8 power thermal scaling under laminar and turbulent flows. The heat transfer within a given surface area to and from small colonies are more rapid than to and from large colonies. Therefore, small colonies will have an advantage over large colonies, since they can dissipate heat more effectively through convection. While, our exponents and coefficient values (Table 2) provide a range of thermal performance under a range of flow velocities and regimes, there remains limited amount of experimental work on to further validate our predictions here. This study along with our previous investigation [42] indicates that total heat loss should increase with increasing surface area and decrease with increasing volume. The results suggest that the temporal response of corals exposed to fluctuating irradiance was affected by coral shape and/or size (*A*/*V* ratios). We previously suggested that since the time constant *τ* is proportional to coral size, the time until the maximum temperature is reached at equilibrium is considerably shorter for smaller corals. Massive or hemispherical corals were previously observed to take nearly twice as long to equilibrate. Hence, the effect of the time constant *τ* on the cooling curve depends strongly on coral size and/or shape (*A*/*V* ratios). Thus, the smaller the time constant *τ*, the faster a coral heats up after the onset of irradiance fluctuations. This is evidence that coral bleaching prediction should not rely simply upon based on predictive modelling, and therefore it is essential, to include the hydrodynamic and heat (and mass) transfer modelling to better describe the bleaching response.

The approximation we have developed here can be used to predict heat exponents and allometric constants related to variation in coral morphologies (*A*/*V* ratios), water-flow velocities (*Re*), and levels of irradiance (*I*). As such, this study presents the first approximation of coral surface warming under both laminar and turbulent flow regimes based on an energy budget analysis. Our numerical predictions agreed well with experimental results (Figs 6 and 7), implying that heat exponents and allometric exponents for both massive or branching colonies can influence the susceptibility of microscale temperature differences detected among species and different colony sizes. Temperature differences of coral surfaces can be associated with convective heat transfer which is a function of the thermal boundary layer. For a given irradiance (*I*), the slope of the relationship between microscale warming and TBL thickness should be proprotional to the coral’s absorptivity and inversely proportional to the thermal conductivity of water and coral (*k*_*m*_ = (1 − *ϕ*)*k*_*c*_ + *k*_*w*_). Therefore, thermal properties of both the tissue and skeleton regions, and morphological traits such as the size and shape of the colony, effect the thermo dynamics regulation of coral microenvironments. Furthermore, the linearised relation observed between predicted and modelled surface warming, provides further validation of the CFD approach adopted here, and the heat and mass transfer theory implemented. Further additional advantage of using this numerical modelling approach is that it can be used directly to predict the thermal coral microenvironment due to variation in flow and light conditions both in experimental chambers and to some extent under field conditions. The physical mechanisms and local forcings found on shallow reef flats due to tidal currents or broken waves can be mimicked by flow patterns in experimental chambers. However, the free-stream parameters applicable to fully turbulent, wave and wind-driven flow on a coral reef—such as turbulence intensity, length scale, and wave dissipation—still need to be fully characterised. Moreover, our models can also be used in conjunction with remotely sensed or in situ observations of irradiance and water flow to produce estimates of coral microscale temperatures. For instance, bleaching is often more severe on shallow reefs [21] during periods of low wind and calm seas, when light penetration is strong [14]. Our validation results and those of Fabricius (2006) establish that microclimatic effects of solar irradiance or light can contribute to increases in the heat load of exposed coral tissue. Both PAR (400–700 nm) and ultraviolet radiation (280–400 nm) have been implicated in triggering coral bleaching [9, 113, 114]. Which waveband contributes more to a coral–s heat budget is likely to depend on the spectral distribution of the light reaching it (the shorter wavelengths carrying higher energy) and the coral tissue absorptivity. Species with high *A*/*V* ratios, branching and plating morphologies, are suited for light interception [115].

It is well established that body temperature is among the most important factors influencing metabolic rate, which in turns reflects a coral’s symbiont ability to convert energy into metabolic products necessary to support movement, growth, maintenance, and reproduction [51, 57]. Strathmann and Strathmann (1982) generalised available hypotheses correlating body size with reproductive mode into three groups, i.e., allometry, dispersal, and variable recruitment; which they found that no single hypothesis explained all known cases [116]. With increasing size, the relative tissue growth rate of massive coral decreases, and the energy which could not be allocated to growth might be channeled to reproduction [117]. Moreover, partial mortality of corals is correlated with colony size [117]. Furthermore, corals are also adversely affected by predation, competition, and physical disturbances; small colonies are often killed outright, while large colonies may survive but sustain substantial loss of tissues [117]. Experimental and observational evidence have indicated that larger corals have lower mortality rates regardless of species [118–120], and that large colonies have high fecundity [121]. Further analyses presented by Gates & Edmunds (1999) [122], Loya *et al*. (2001) [5], and Jimenez *et al*. (2011) [7], suggesting that corals with low growth rates and high metabolic rates, such as massive species, may tolerate greater thermal exposure more or acclimatise more effectively than those with low metabolic rates and fast-growing branching species, corroborate our model-based findings and predictions. Therefore, further investigations is required in order to correlate the interactions between coral physiology and biological properties, which will enhance our understanding of the inter- and intra-specific variation in bleaching susceptibility.

Our research here also illustrates that the relationships between coral morphology, coral thermal microenvironments and a variety of fluctuating environmental conditions are both labile and complex. At the scale of individual colonies, the interaction of coral morphology with the surrounding flow combined with regular exposure to irradiance, together with differences in surface area-to-volume ratios, could affect the heat transfer between the coral and the surrounding water, and thus influence the temperature of the tissue. The extent to which these processes can help explain size- and shape-related patterns of coral bleaching in nature requires further investigation. Such future research focused on the dynamics of microscale surface temperature would help satisfy, the need for an experimental measurements to validate this relationship. Future work should also estimate the range of thermal conductivity values for tissue and skeleton. Finally, the current CFD model could be used in conjunction with detailed biophysical modelling of coral growth and morphogenesis in order to better understand processes underpinning allometric scaling in colonial modular taxa.

## Summary

In summary, this study explores allometric scaling of the thermal dynamics of coral colonies with highly divergent morphologies. Our results show that although corals may be exposed to a uniform irradiance and flow velocities, differential effects of coral morphology may cause some corals to exceed critical surface temperatures more than others. Using the methods presented here, however, the heat exposure of corals at the scale of individual colonies can be estimated with reasonable accuracy, and as a consequence, provides reasonably accurate predictions of interspecific differences in surface temperatures. In combination then, this study may assist in identifying thermal stress variability related to intra- and inter-colonial patterns of bleaching. While these novel approximations offer insight into the allometric scaling of coral thermal microenvironments, the values of the constants estimated here remain to be validated via experimentation.

## Supporting information

S1 Text CFD modelling of hydro and thermal physics in coral microenvironments. (PDF)

S2 Text Extended description of the model assemblages and configurations. (PDF)

S3 Text Numerical simulation. (PDF)

S4 TextEffect of flow velocity variations on the *Nu* − *Re* exponent values and the generalised allometric constant $\left(\frac{\Delta T}{Re^{\bar{b^{*}}}}-A/V\right)$ values.(PDF)

S1 Fig Models of the coral species studied here. **(a)** *Acropora millepora*, **(b)** *Diploria labyrinthiformis*, **(c)** generalised massive coral, **(d)** *Seriatopora hystrix*, **(e)** generalised *Fungia sp.*, **(f)** *Seriatopora caliendrum*, **(g)** generalised *Goniastrea aspera*, **(h)** generalised section of a cylindrical coral branch, **(i)** *Montastrea annularis*, **(j)** *Madracis mirabilis*, **(k)** *Porites* sp, **(l)** *Acropora digitifera*.
(EPS)

S2 Fig Conceptual representation of the model setup used in the simulation at constant flow (0.01 m s^−1^) and irradiance of 650 W m^−2^. (EPS)

S3 Fig *Nu* − *Re* plots of the whole assemblage (combination of branching (B) and massive (M) colonies) in both laminar and turbulent regimes. (EPS)

S4 Fig *Nu* − *Re* plots of branching colony (B) and massive colony (M) showing different flow velocities in laminar regime. (EPS)

S5 Fig *Nu* − *Re* plots of branching colony (B) and massive colony (M) showing different flow velocities in turbulent regime. (EPS)

S6 FigGeneralised allometric model constants $\left(\frac{\Delta T}{Re^{\bar{b^{*}}}}-A/V\right)$ plots of whole assemblage (combination of branching (B) and massive (M)) of both laminar and turbulent regimes at a constant flow velocity of 0.01 m s^−1^.(EPS)

S7 FigGeneralised allometric model constants $\left(\frac{\Delta T}{Re^{\bar{b^{*}}}}-A/V\right)$ plots of branching colony (B) and massive colony (M) within the laminar regime at flow velocities of 0.01, 0.002, and 0.001 m s^−1^.(EPS)

S8 FigGeneralised allometric model constants $\left(\frac{\Delta T}{Re^{\bar{b^{*}}}}-A/V\right)$ plots of branching colony (B) and massive colony (M) within the turbulent regime at flow velocities of 0.01 and 0.001 m s^−1^.(EPS)

S9 Fig Relationships between Δ*T* for simulated against numerical predictions for the whole assemblage (combination of massive (M) and branching (B) colonies) in both laminar and turbulent regimes. (EPS)

S1 Table List of symbols, their dimensions, and definitions. (PDF)

S2 Table Model assemblages and predicted similarity ratios, where B and M denote branching and massive morphologies, respectively. *A* and *V* denote area and volume.
(PDF)

S3 Table List of steady-state simulation runs performed at a constant water velocity of 0.01 m s^−1^ exposed to sunlight of ∼650 W m^−2^. (PDF)

S4 Table List of steady-state simulation runs performed at varying water flow velocities (1-10 cm s^−1^) exposed to sunlight of ∼650 W m^−2^. (PDF)

S5 Table Near-wall damping terms and free-stream parameters, where *C*_*μ*_ and *β*_1_ represent constant values of 0.09 and 0.075, respectively. (PDF)

S6 Table Initial and boundary conditions for laminar and turbulent simulations, where S: Slip, ZG: Zero Gradient, ZNG: Zero Normal Gradient, FV: Fixed Value, IO: Input Output, TIKEI: Turbulent Intensity Kinetic Energy Inlet, TMLFI: Turbulent Mixing Length Frequency Inlet. (PDF)

S7 Table Summary of local heat coefficients (*a*) and exponents (*b*) based on the colony shape and the allometric thermal scaling constants for both the laminar (L) and turbulent (T) regimes. (PDF)
