# Supplementary material for: The effect of allometric scaling in coral thermal microenvironments
Source: PLoS One. 2017 Oct 12;12(10):e0184214. doi: 10.1371/journal.pone.0184214 (PMC5638381; doi:10.1371/journal.pone.0184214)
Supplement: S1 Text — (PDF) [file pone.0184214.s001.pdf]

The Effect of Allometric Scaling in Coral Thermal Microenvironments

Robert H. Ong[1,†], Andrew J.C. King[1], Jaap A. Kaandorp[2], Benjamin J. Mullins[1,3,*], M. Julian Caley[4,5]

1. Fluid Dynamics Research Group, Curtin Institute for Computation, Department of Mechanical Engineering, Curtin University, Perth, Australia
2. Computational Science Section, University of Amsterdam, Amsterdam, The Netherlands
3. Occupation and Environment, School of Public Health, Curtin University, Perth, Australia
4. School of Mathematical Sciences, Queensland University of Technology, Brisbane, Queensland, Australia
5. Australian Research Council Centre of Excellence for Mathematical and Statistical Frontiers

† **Present address: Centre for Wind, Waves and Water, School of Civil Engineering, The University of Sydney, NSW, Australia**

* **Corresponding author email: b.mullins@curtin.edu.au**

Supporting Information

S1 Text

CFD modelling of hydro and thermal physics in coral microenvironments

The physical laws governing porous media, fluid flow, and heat transfer processes must firstly be defined as differential equations. Expressions of each symbol and dimension and their definitions are listed in S1 Table.

Mass continuity equation:

$$\frac{\partial u}{\partial x} + \frac{\partial v}{\partial y} + \frac{\partial w}{\partial z} = 0 \tag{1}$$

Momentum equation:

$$u\frac{\partial u}{\partial x} + v\frac{\partial v}{\partial y} + w\frac{\partial w}{\partial z} = -\frac{\partial p}{\partial x} + \nu\left(\frac{\partial^2 u}{\partial x^2} + \frac{\partial^2 u}{\partial y^2} + \frac{\partial^2 u}{\partial z^2}\right) - S_{ix} \tag{2}$$

$$u\frac{\partial u}{\partial x} + v\frac{\partial v}{\partial y} + w\frac{\partial w}{\partial z} = -\frac{\partial p}{\partial y} + \nu\left(\frac{\partial^2 v}{\partial x^2} + \frac{\partial^2 v}{\partial y^2} + \frac{\partial^2 v}{\partial z^2}\right) - S_{iy} \tag{3}$$

$$u\frac{\partial u}{\partial x} + v\frac{\partial v}{\partial y} + w\frac{\partial w}{\partial z} = -\frac{\partial p}{\partial z} + \nu\left(\frac{\partial^2 w}{\partial x^2} + \frac{\partial^2 w}{\partial y^2} + \frac{\partial^2 w}{\partial z^2}\right) - S_{iz} \tag{4}$$

where ∂u, ∂v, and ∂w represent velocity components of each axis-of-coordinates direction, ν is kinematic viscosity of the fluid (water), p is reference pressure, α is coral absorptivity, Q is the heat irradiance. The flow sink term, S_i is composed of two parts,

a viscous loss term and inertial loss term, creating a pressure drop that is proportional to the velocity and square of the velocity, respectively:

$$S_i = -(\nu\, D_i + \frac{1}{2}\,|\mathbf{U}|\, F_i)\mathbf{U} \tag{5}$$

where D_i and F_i are represented as the scalars D and F, known as the Darcy-Forchheimer coefficients (refer below to Equation 13).

The mathematical model for steady, turbulent incompressible flow consists of the Reynolds average Navier-Stokes equation which solves for the mean velocity and pressure values and can be written as:

$$\frac{\partial}{\partial x_j}\left(\bar{u_j}\,\bar{u_i}\right) = -\frac{\partial}{\partial x_i}\left(\frac{\bar{p}}{\rho}\right) + \frac{1}{\rho}\,\frac{\partial}{\partial x_j}\left(\tau_{ij} + \tau_{t_{ij}}\right) - S_i \tag{6}$$

The vectors u_i and x_i are velocity and position, $\tau_{t_{ij}}$ is the Reynolds-stress tensor, and τ_{ij} is the mean laminar viscous stress and is given by:

$$\tau_{ij} = \mu\left[\left(\frac{\partial \bar{u_i}}{\partial x_j} + \frac{\partial \bar{u_j}}{\partial x_i}\right) - \frac{2}{3}\left(\frac{\partial \bar{u_k}}{\partial x_k}\right)\delta_{ij}\right] \tag{7}$$

where δ_{ij} is unit tensor. The Reynolds-stress tensor represents the transfer of momentum due to turbulent fluctuations, can be written as:

$$\tau_{t_{ij}} = 2\mu_T\, S_{ij} - \frac{2}{3}\,\rho\, k\, \delta_{ij} \tag{8}$$

where S_{ij} is the mean strain-rate tensor and k is turbulent kinetic energy which is given as:

$$k = \frac{1}{2}\bar{u_i}'\,\bar{u_i}' = \frac{1}{2}\left(\bar{u}'^2 + \bar{v}'^2 + \bar{w}'^2\right) \tag{9}$$

The degree of turbulence can be adequately represented using the intensity of turbulence, I, and the scale of turbulence, l (described in later section).

Energy equation:

$$\frac{\partial}{\partial x_j}\left(\bar{T}\bar{u_j}\right) = \frac{\partial}{\partial x_k}\left(\Gamma_{T_{eff}}\,\frac{\partial \bar{T}}{\partial x_k}\right) + \frac{\alpha\, Q}{\rho\, C_p} + \bar{\Phi} \tag{10}$$

where $\Gamma_{T_{eff}}$ is the effective thermal diffusivity $\left(\frac{\nu_0}{Pr} + \frac{\nu_t}{Pr_t}\right)$, and Φ is turbulent heat dissipation fluxes $\left[\frac{\mu}{2}\left(\frac{\partial \bar{u_i}}{\partial x_j} + \frac{\partial u_i'}{\partial x_j} + \frac{\partial \bar{u_j}}{\partial x_i} + \frac{\partial u_j'}{\partial x_i}\right)^2\right]$, which has an effect on the temperature rise only for cases with high flow velocity gradients. Previous observations [1] showed that the low-Reynolds turbulence $k-\omega$ Shear Stress Transport (SST) model agreed well with experimental data [2], and tended to establish downstream laminar flow when the free-stream turbulence level was low $\left(\frac{\mu_t}{\mu} \leq 0.1\right)$. Further details of the $k-\omega$ SST model can be found in [3–5].

Fluid flow and heat transfer through a porous coral

The change of pressure drop can be expressed using the Darcy-Forchheimer's equation:

$$\frac{\partial P}{\partial x} = -\frac{\beta\mu(1-\phi)^2\mathbf{U}}{D^2\phi^3} - \frac{\delta\rho_f(1-\phi)\mathbf{U}^2}{D\phi^3} \tag{11}$$

where $\mathbf{U}$ is a velocity vector, ϕ is porosity, D is the mean diameter of the medium, μ and ρ are dynamic vicosity and density of the fluid, and β and δ are empirical shape

factors. A general form of pressure drop across a porous medium in an unidirectional flow can be determined using Ergun's equation (with $\delta = 1.75$ and $\beta = 150$), consisting of two parts: a viscous loss term and intertial loss term, creating a pressure drop that is proportional to the velocity and square of velocity, respectively. The first term of the friction factor (referred to as Darcy's coefficient) given by the Carman-Kozeny equation specifies viscous energy primarily at low Reynolds number or laminar flow, and the second term of the friction factor (referred to Forchheimer's coefficient) represented by Burke-Plummer equation describes kinetic energy primarily at larger Reynolds number or turbulent. These can be written as:

$$D = \frac{150(1-\phi)^2}{L_c^2\phi^3} \tag{12}$$

$$F = \frac{1.75(1-\phi)}{L_c\phi^3} \tag{13}$$

From the heat transfer theory, assuming that there is local thermal equilibrium so that $T_s = T_f = T$, where T_s and T_f are the temperatures of the solid and fluid phases, respectively. Averaged material properties for the (coral) porous media can be written as:

$$\rho_{eff} = (1-\phi)\rho_c + \phi\rho_f \tag{14}$$

$$C_{p_{eff}} = (1-\phi)C_{p_c} + \phi C_{p_f} \tag{15}$$

$$k_{eff} = (1-\phi)k_c + \phi k_f \tag{16}$$

where equations 14, 15, and 16 represent effective density, effective heat capacity, and effective thermal conductivity of the medium, respectively. These values were then used to calculate the effective thermal diffusivity of the medium, Γ_{T_m}, which is given by:

$$\Gamma_{T_m} = \frac{k_m}{(\rho C_p)_m} \tag{17}$$

References

1. Ong RH, King ACJ, Mullins BJ, Caley MJ, Cooper TF. CFD Simulation of Low Reynolds-number Turbulence Models in Coral Thermal Microenvironment. In: Australian Fluid Mechanics Society; 2012. .

2. Fabricius KE. Effects of irradiance, flow, and colony pigmentation on the temperature microenvironment around corals: Implications for coral bleaching? Limnology and Oceanography. 2006;51(1):30–37.

3. Menter FR. Two-equation eddy-viscosity turbulence models for engineering applications. AIAA journal. 1994;32(8):1598–1605.

4. Wilcox DC, et al. Turbulence modeling for CFD. vol. 2. DCW industries La Canada, CA; 1998.

5. Menter F, Kuntz M, Langtry R. Ten years of industrial experience with the SST turbulence model. Turbulence, heat and mass transfer. 2003;4:625–632.
