# Supplementary material for: The effect of allometric scaling in coral thermal microenvironments
Source: PLoS One. 2017 Oct 12;12(10):e0184214. doi: 10.1371/journal.pone.0184214 (PMC5638381; doi:10.1371/journal.pone.0184214)
Supplement: S2 Text — (PDF) [file pone.0184214.s002.pdf]

The Effect of Allometric Scaling in Coral Thermal Microenvironments

Robert H. Ong[1,†], Andrew J.C. King[1], Jaap A. Kaandorp[2], Benjamin J. Mullins[1,3,*], M. Julian Caley[4,5]

1. Fluid Dynamics Research Group, Curtin Institute for Computation, Department of Mechanical Engineering, Curtin University, Perth, Australia
2. Computational Science Section, University of Amsterdam, Amsterdam, The Netherlands
3. Occupation and Environment, School of Public Health, Curtin University, Perth, Australia
4. School of Mathematical Sciences, Queensland University of Technology, Brisbane, Queensland, Australia
5. Australian Research Council Centre of Excellence for Mathematical and Statistical Frontiers

† **Present address: Centre for Wind, Waves and Water, School of Civil Engineering, The University of Sydney, NSW, Australia**

* **Corresponding author email: b.mullins@curtin.edu.au**

Supporting Information

S2 Text

Extended description of the model assemblages and configurations

Most of the specified coral shapes studied here were scanned using a medical computed-tomography (CT) scanner with isotropic resolution. We reconstructed the scans using an image processing package, Fiji [1], converted these reconstructions to a binary stereo lithography (STL) format, and visualised them using the visualisation toolkit software. A few additional generalised models were manually reconstructed with the aid of computer-aided-design (CAD) software (Blender [2]). From a morphological standpoint, corals can be classed into two main shape categories: (1) branching forms with mostly large branching, tabulate, or corymbose morphologies; and (2) massive forms with mostly massive, columnar, foliose, or encrusting morphologies (S2 Table and S1 Fig). The scaling properties of these forms do not depend on most of the details of their morphologies, such as details of their branching patterns. The characteristic dimension, L, was taken to be equal to the height, normal to the substrate. Heights, diameters and lengths were measured in meters. A dimensionless shape index was calculated from surface area$^{0.5}$ $\times$ volume$^{-0.33}$ [3]. The degree of overall similarity between branching and massive coral morphologies (the ratio K) was evaluated using:

$$\frac{V}{A} \propto L \quad \text{or} \quad \frac{V}{A} = \frac{k}{k'}\, L = K\, L \tag{1}$$

where k, k' are similarity ratios or factors of proportion [4].

References

1. Schindelin J, Arganda-Carreras I, Frise E, Kaynig V, Longair M, Pietzsch T, et al. Fiji: an open-source platform for biological-image analysis. Nature methods. 2012;9(7):676–682.

2. Foundation B. Blender Manual; 2015. Version 2.75, https://www.blender.org/features/2-75/.

3. Vogel S. Life in moving fluids: the physical biology of flow. Princeton University Press; 1981.

4. Schmidt-Nielsen K. Scaling: why is animal size so important? Cambridge University Press; 1984.
