# Supplementary material for: The effect of allometric scaling in coral thermal microenvironments
Source: PLoS One. 2017 Oct 12;12(10):e0184214. doi: 10.1371/journal.pone.0184214 (PMC5638381; doi:10.1371/journal.pone.0184214)
Supplement: S3 Text — (PDF) [file pone.0184214.s003.pdf]

The Effect of Allometric Scaling in Coral Thermal Microenvironments

Robert H. Ong[1,†], Andrew J.C. King[1], Jaap A. Kaandorp[2], Benjamin J. Mullins[1,3,*], M. Julian Caley[4,5]

1. Fluid Dynamics Research Group, Curtin Institute for Computation, Department of Mechanical Engineering, Curtin University, Perth, Australia
2. Computational Science Section, University of Amsterdam, Amsterdam, The Netherlands
3. Occupation and Environment, School of Public Health, Curtin University, Perth, Australia
4. School of Mathematical Sciences, Queensland University of Technology, Brisbane, Queensland, Australia
5. Australian Research Council Centre of Excellence for Mathematical and Statistical Frontiers

† **Present address: Centre for Wind, Waves and Water, School of Civil Engineering, The University of Sydney, NSW, Australia**

* **Corresponding author email: b.mullins@curtin.edu.au**

Supporting Information

S3 Text

Numerical simulation

S3 Table and S4 Table present simulation runs performed with constant and varying flow velocities which provided insights to the effects of coral size and shape on thermal dynamics (dimensionless $Nu - Re$ relationship in Equation 10 of the main article. The numerical schemes of SIMPLE (semi implicit method for pressure-linked equations) were used to resolve the pressure velocity coupling at each iteration for the steady-state simulations. This study estimated both the surface area and volume of coral 3D models using a cell looping technique that calculates and increments cell area and volume over a mesh-based representation of complex morphologies [2]. There are two types of grids used here; block structured hexahedral and polyhedral meshes, to represent both fluid and coral computational domains. For laminar flow simulations, only 900,000 cells were required to satisfy mesh independence. Our previous study showed that the grids used in our models were sufficiently fine to capture these heating dynamics, and further refinement of the grid would have been unlikely to yield significant variations to our results [2].

The low-Reynolds $k - \omega$ Shear Stress Transport model was used, in which turbulence was damped due to near-wall regions or low permeability porous media. The wall regions were resolved using boundary conditions for the first grid point near the wall, wherein the viscous sublayer region was accounted. The dimensionless quantity of normalised distance from the wall (y^+ is defined in S5 Table). Here, the y$^+$ values were maintained at approximately 2.0 with ∼2,500,000 cells, required to produce results

independent of the mesh size. To force the correct near-wall behavior, it is necessary to specify the initial conditions and make an educated judgment for the mean value of free-stream turbulence parameters, shown in S5 Table. We estimated the turbulence intensity (I) and length scale (l_{sc}) to be ∼1% and ∼30% of coral characteristic length, respectively [1]. To represent flow conditions corresponding to a typical flow chamber, it was necessary to define initial and boundary conditions of the computational domain. For both velocity and turbulent kinetic energy no-slip condition stands at the wall. The pressure at the inlet was fixed at the zero gradient condition. The pressure at the outlet were fixed at the reference pressure, while the outlet velocity was fixed at the zero gradient. The initial and boundary conditions for laminar and turbulent simulations are shown in S6 Table.

References

1. Ong RH, King ACJ, Mullins BJ, Caley MJ, Cooper TF. CFD Simulation of Low Reynolds-number Turbulence Models in Coral Thermal Microenvironment. In: Australian Fluid Mechanics Society; 2012. .

2. Ong RH, King AJC, Mullins BJ, Cooper TF, Caley MJ. Development and Validation of Computational Fluid Dynamics Models for Prediction of Heat Transfer and Thermal Microenvironments of Corals. PLoS One. 2012;7(6):e37842.
