# Supplementary material for: The effect of allometric scaling in coral thermal microenvironments
Source: PLoS One. 2017 Oct 12;12(10):e0184214. doi: 10.1371/journal.pone.0184214 (PMC5638381; doi:10.1371/journal.pone.0184214)
Supplement: S4 Text — (PDF) [file pone.0184214.s004.pdf]

The Effect of Allometric Scaling in Coral Thermal Microenvironments

Robert H. Ong[1,†], Andrew J.C. King[1], Jaap A. Kaandorp[2], Benjamin J. Mullins[1,3,*], M. Julian Caley[4,5]

1. Fluid Dynamics Research Group, Curtin Institute for Computation, Department of Mechanical Engineering, Curtin University, Perth, Australia
2. Computational Science Section, University of Amsterdam, Amsterdam, The Netherlands
3. Occupation and Environment, School of Public Health, Curtin University, Perth, Australia
4. School of Mathematical Sciences, Queensland University of Technology, Brisbane, Queensland, Australia
5. Australian Research Council Centre of Excellence for Mathematical and Statistical Frontiers

† **Present address: Centre for Wind, Waves and Water, School of Civil Engineering, The University of Sydney, NSW, Australia**

* **Corresponding author email: b.mullins@curtin.edu.au**

Supporting Information

S4 Text

Effect of flow velocity variations on the $Nu - Re$ exponent values and the generalised allometric constant $\left(\frac{\Delta T}{Re^{b*}} - A/V\right)$ values.

The influence of various water velocities (from 1-10 cm s^{-1}) in laminar (S4 Fig) and turbulent flows (S5 Fig) established that as the Reynolds number increases, the average Nusselt number also increases. The large Reynolds number was due to the higher velocity which can lead to flow disturbance and thus, higher heat transfer augmentation. This augmentation can partly be explained by a stronger turbulence intensity generated by greater water velocity, leading to a rapid mixing of flow especially at higher Reynolds numbers.

Log-log scatter plots of allometric thermal scaling of various coral shapes and sizes varied between 1 and 10 cm s^{-1} for both the laminar S7 Fig and turbulent flows S8 Fig, provided similar values in terms of the scaling exponents (m). The slopes of thermal allometric plots indicated a directly proportional relationship for the branching colony and an inversely proportional relationship for the massive colony, suggesting that the microscale temperature of massive corals increased disproportionately slower under the turbulent flow.

The derived heat exponents and allometric constants for various colony shapes at constant flow of 10 cm s^{-1} is given in S7 Table. The scatterplots of whole assembalge (combination of massive **(M)** and branching **(B)** colonies) between ΔT for simulated against numerical predictions in both laminar and turbulent regimes (S9 Fig), found

some bias thereby emphasising the role of coral shape in the overall microscale's surface temperature distributions.
