# Supplementary material for: The effect of allometric scaling in coral thermal microenvironments
Source: PLoS One. 2017 Oct 12;12(10):e0184214. doi: 10.1371/journal.pone.0184214 (PMC5638381; doi:10.1371/journal.pone.0184214)
Supplement: S1 Table — (PDF) [file pone.0184214.s014.pdf]

S1 Table

List of symbols, their dimensions, and definitions.

α [-]	**tissue absorptivity**	h $[Wm^{-2}K^{-1}]$	**heat transfer coefficient**
A $[m^2]$	**coral surface area**	I $[Wm^2]$	**irradiance**
T_t $[K]$	**coral tissue temperature**	T_f $[K]$	**ambient fluid temperature**
$\mathbf{U}$ $[ms^{-1}]$	**superficial velocity vector**	ϕ [%]	**porosity**
ν $[m^2s^{-1}]$	**kinematic viscosity**	μ $[Nsm^{-2}]$	**dynamic viscosity**
ρ_c $[kgm^3]$	**coral density**	ρ_f $[kgm^3]$	**fluid density**
C_{p_c} $[Jkg^{-1}K^{-1}]$	**coral heat capacity**	C_{p_f} $[Jkg^{-1}K^{-1}]$	**fluid heat capacity**
k_c $[Wm^{-1}K^{-1}]$	**coral thermal conductivity**	k_f $[Wm^{-1}K^{-1}]$	**fluid thermal conductivity**
Γ_{T_m} $[m^2s^{-1}]$	**medium thermal diffusivity**	Pr [-]	**Prandtl number**
D $[ms^{-1}]$	**Darcy's term**	F $[ms^{-1}]$	**Forchheimer's term**
k $[m^2s^{-2}]$	**turbulent kinetic energy**	ω $[m^2s^{-3}]$	**specific dissipation rate**
