# Supplementary material for: The effect of allometric scaling in coral thermal microenvironments
Source: PLoS One. 2017 Oct 12;12(10):e0184214. doi: 10.1371/journal.pone.0184214 (PMC5638381; doi:10.1371/journal.pone.0184214)
Supplement: S2 Table — A and V denote area and volume. (PDF) [file pone.0184214.s015.pdf]

S2 Table

Model assemblages and predicted similarity ratios, where B and M denote branching and massive morphologies, respectively. A and V denote area and volume.

Species	shape categories	characteristic length (L), m	similarity ratio (K)	shape index $A^{0.5} \times V^{-0.33}$
Acropora digitifera	B	0.483	45	10.95
Acropora millepora	B	0.559	42	10.95
Diploria labyrinthiformis	M	0.906	50	13.12
generalised massive coral	M	0.525	1.2	2.79
generalised *Fungia* sp.	M	0.790	73	12.55
generalised cylindrical branch	B	0.450	66	7.64
generalised *Goniastrea aspera*	M	0.397	1.5	2.87
Montastrea annularis	B	0.480	40	12
Madracis mirabilis	B	0.488	43	10.20
Porites sp.	M	0.64	53	11.42
Seriatopora caliendrum	B	0.56	38	11.22
Seriatopora hystrix	B	0.49	236	8.92
