# Supplementary material for: The effect of allometric scaling in coral thermal microenvironments
Source: PLoS One. 2017 Oct 12;12(10):e0184214. doi: 10.1371/journal.pone.0184214 (PMC5638381; doi:10.1371/journal.pone.0184214)
Supplement: S3 Table — (PDF) [file pone.0184214.s016.pdf]

## S3 Table

**List of steady-state simulation runs performed at a constant water velocity of 0.01 m $s^{-1}$ exposed to sunlight of ∼650 W $m^{-2}$.**

| Species | Laminar | | Turbulent | |
|---|---|---|---|---|
| | L (m) | Re | L (m) | Re |
| *A. digitifera* (B) | 0.00483 | 45 | 0.483 | 4472 |
| | 0.02415 | 224 | 2.415 | 22361 |
| | 0.0483 | 447 | 4.83 | 44722 |
| *A. millepora* (B) | 0.0056 | 52 | 0.56 | 5178 |
| | 0.028 | 259 | 2.80 | 25893 |
| | 0.056 | 518 | 5.6 | 51787 |
| *D. labyrinthiformis* (M) | 0.091 | 839 | 0.906 | 8392 |
| | 0.0453 | 419 | 4.532 | 41962 |
| | 0.0091 | 84 | 9.06 | 83925 |
| generalised massive (M) | 0.0053 | 49 | 0.525 | 4861 |
| | 0.0263 | 243 | 2.625 | 24305 |
| | 0.0525 | 486 | 5.25 | 48611 |
| *Fungia* sp. (M) | 0.0079 | 73 | 0.79 | 7314 |
| | 0.0395 | 365 | 3.95 | 36574 |
| | 0.079 | 731 | 7.9 | 73148 |
| cylindrical branch (B) | 0.0045 | 42 | 0.45 | 4167 |
| | 0.0225 | 292 | 2.25 | 20833 |
| | 0.0045 | 417 | 4.5 | 41667 |
| *G. aspera* (M) | 0.004 | 37 | 0.4 | 3681 |
| | 0.02 | 184 | 2 | 18407 |
| | 0.04 | 368 | 4 | 36814 |
| *M. annularis* (B) | 0.0048 | 44 | 0.48 | 4444 |
| | 0.024 | 222 | 2.4 | 22222 |
| | 0.048 | 444 | 4.8 | 44444 |
| *M. mirabilis* (B) | 0.0049 | 45 | 0.488 | 4518 |
| | 0.0244 | 226 | 2.44 | 22592 |
| | 0.0488 | 452 | 4.88 | 45185 |
| *Porites* sp. (M) | 0.064 | 60 | 0.644 | 5963 |
| | 0.0322 | 298 | 3.22 | 29815 |
| | 0.0644 | 596 | 6.44 | 59630 |
| *S. caliendrum* (B) | 0.0056 | 52 | 0.56 | 5185 |
| | 0.0112 | 103 | 2.8 | 25925 |
| | 0.056 | 518 | 5.6 | 51851 |
| *S. hystrix* (B) | 0.0049 | 45 | 0.49 | 4537 |
| | 0.0245 | 227 | 2.45 | 22685 |
| | 0.049 | 454 | 4.90 | 45370 |
