# Supplementary material for: The effect of allometric scaling in coral thermal microenvironments
Source: PLoS One. 2017 Oct 12;12(10):e0184214. doi: 10.1371/journal.pone.0184214 (PMC5638381; doi:10.1371/journal.pone.0184214)
Supplement: S4 Table — (PDF) [file pone.0184214.s017.pdf]

S4 Table

List of steady-state simulation runs performed at varying water flow velocities (1-10 cm s^{-1}) exposed to sunlight of ∼650 W m^{-2}.

Species	Laminar			Turbulent		
	L (m)	U (m/s)	Re	L (m)	U (m/s)	Re
A. digitifera (B)	0.0345	0.01	345	4.9	0.01	4.54×10^4
	0.1725	0.002	345	-	-	-
	0.345	0.001	345	49	0.001	4.54×10^4
A. millepora (B)	0.074	0.01	740	5.6	0.01	5.19×10^4
	0.37	0.002	740	-	-	-
	0.74	0.001	740	56	0.001	5.19×10^4
D. labyrinthiformis (M)	0.085	0.01	850	1.8	0.01	1.67×10^4
	0.425	0.002	850	-	-	-
	0.85	0.001	850	18	0.001	1.67×10^4
generalised massive (M)	0.011	0.01	110	3.5	0.01	3.24×10^4
	0.055	0.002	110	-	-	-
	0.11	0.001	110	35	0.001	3.24×10^4
Fungia sp. (M)	0.079	0.01	790	7.904	0.01	7.32×10^4
	0.395	0.002	790	-	-	-
	0.79	0.001	790	79.04	0.001	7.32×10^4
cylindrical branch (B)	0.12	0.01	1200	12	0.01	1.11×10^5
	0.6	0.002	1200	-	-	-
	1.2	0.001	1200	120	0.001	1.11×10^5
G. aspera (M)	0.01	0.01	112	4	0.01	5.19×10^4
	0.05	0.002	112	-	-	-
	0.11	0.001	112	40	0.001	5.19×10^4
M. annularis (B)	0.032	0.01	320	0.714	0.01	6.61×10^3
	0.16	0.002	320	-	-	-
	0.32	0.001	320	7.14	0.001	6.61×10^3
M. mirabilis (B)	0.0022	0.01	22	7.32	0.01	6.78×10^4
	0.011	0.002	22	-	-	-
	0.022	0.001	22	73.2	0.001	6.78×10^4
Porites sp. (M)	0.01	0.01	100	8.2	0.01	7.59×10^4
	0.05	0.002	100	-	-	-
	0.1	0.001	100	82	0.001	7.59×10^4
S. caliendrum (B)	0.015	0.01	150	5.6	0.01	5.19×10^4
	0.075	0.002	150	-	-	-
	0.15	0.001	150	56	0.001	5.19×10^4
S. hystrix (B)	0.015	0.023	230	5	0.01	4.63×10^4
	0.115	0.002	230	-	-	-
	0.23	0.001	230	50	0.001	4.63×10^4
