# Supplementary material for: The effect of allometric scaling in coral thermal microenvironments
Source: PLoS One. 2017 Oct 12;12(10):e0184214. doi: 10.1371/journal.pone.0184214 (PMC5638381; doi:10.1371/journal.pone.0184214)
Supplement: S5 Table — (PDF) [file pone.0184214.s018.pdf]

## S5 Table

**Near-wall damping terms and free-stream parameters**, where $C_\mu$ and $\beta_1$ represent constant values of 0.09 and 0.075, respectively.

| Parameter | Formulae |
|---|---|
| normalised distance from the wall ($y^+$) | $\frac{y \cdot u_\star}{\nu}$ |
| friction velocity ($u_\star$) | $\sqrt{\frac{\tau_w}{\rho}}$ |
| wall shear stress ($\tau_w$) | $\frac{C_f \rho {U_0}^2}{2}$ |
| skin friction coefficient ($C_f$) | $\frac{0.0791}{Re^{-0.3}}$ |
| turbulent kinetic energy ($k$) [$m^2$ $s^{-2}$] | $\frac{3}{2}\left(I\bar{U}\right)^2$ |
| turbulent dissipation rate ($\epsilon$) [$m^2$ $s^{-3}$] | $\frac{C_\mu^{3/4} k^{3/2}}{l_{sc}}$ |
| turbulent specific dissipation rate ($\omega$) [$s^{-1}$] | ${C_\mu}^{-\frac{1}{4}} \frac{\sqrt{k}}{l}$ |
| wall turbulent specific dissipation rate ($\omega_{wall}$) [$s^{-1}$] | $\frac{60\,\nu}{\beta_1\, y^2}$ |
