# Supplementary material for: The effect of allometric scaling in coral thermal microenvironments
Source: PLoS One. 2017 Oct 12;12(10):e0184214. doi: 10.1371/journal.pone.0184214 (PMC5638381; doi:10.1371/journal.pone.0184214)
Supplement: S6 Table — (PDF) [file pone.0184214.s019.pdf]

S6 Table

Initial and boundary conditions for laminar and turbulent simulations, where S: Slip, ZG: Zero Gradient, ZNG: Zero Normal Gradient, FV: Fixed Value, IO: Input Output, TIKEI: Turbulent Intensity Kinetic Energy Inlet, TMLFI: Turbulent Mixing Length Frequency Inlet.

Flow type	Parameter	Initial Value	Surrounds	Seafloor	Inlet	Outlet
Laminar	reference pressure (p_{ref})	0 Pa	S	ZG	ZG	FV
	velocity (u)	0.01 m s^{-1}	S	(0 0 0)	FV	ZNG
	temperature (T)	299 K	S	FV	FV	ZG
Turbulent	turbulent kinetic energy (k)	10^{-10} m^2 s^{-2}	ZG	TIKEI	IO	ZG
	turbulent specific dissipation rate (ω)	5×10^{-4} s^{-1}	ZG	TMLFI	IO	ZG
