# Supplementary material for: The effect of allometric scaling in coral thermal microenvironments
Source: PLoS One. 2017 Oct 12;12(10):e0184214. doi: 10.1371/journal.pone.0184214 (PMC5638381; doi:10.1371/journal.pone.0184214)
Supplement: S7 Table — (PDF) [file pone.0184214.s020.pdf]

S7 Table

Summary of local heat coefficients (a) and exponents (b) based on the colony shape and the allometric thermal scaling constants for both the laminar (L) and turbulent (T) regimes.

Shape	Local a and b		Local allometric constants	
	a	b	C	m
Flat plate or mushroom (L)	0.68	0.82	6.45×10^{-3}	0.12
Flat plate or mushroom (T)	1.16	0.95	1.17×10^{-4}	0.40
Cylinder (L)	0.96	0.68	1.14×10^{-3}	0.48
Cylinder (T)	0.68	0.87	7.94×10^{-6}	0.74
masive - hemispherical (L)	0.83	0.56	5.75×10^{-3}	0.28
massive - hemispherical (T)	1.52	0.75	1.35×10^{-4}	0.36
massive - *G. aspera* (L)	0.75	0.47	1.18×10^{-1}	-0.16
massive - *G. aspera* (T)	0.96	0.83	1.99×10^{-4}	-0.09
massive - *Porites* (L)	0.89	0.53	1.82×10^{-1}	-0.16
massive - *Porites* (T)	1.63	0.69	2.51×10^{-4}	0.22
encrusting - *D. labyrinthiformis* (L)	0.83	0.55	1.54×10^{-2}	0.14
encrusting - *D. labyrinthiformis* (T)	1.10	0.86	2.51×10^{-5}	0.57
columnar - *S. hystrix* (L)	0.79	0.58	4.57×10^{-3}	0.36
columnar - *S. hystrix* (T)	0.15	1.16	7.94×10^{-11}	1.12
columnar - *S. caliendrum* (L)	0.80	0.50	1.99×10^{-2}	0.23
columnar - *S. caliendrum* (T)	0.89	0.94	1.00×10^{-5}	0.80
tabular - *A. millepora* (L)	0.58	0.57	3.98×10^{-3}	0.38
tabular - *A. millepora* (T)	0.98	0.81	1.29×10^{-5}	0.73
digitate - *M. mirabilis* (L)	0.69	0.58	3.71×10^{-3}	0.40
digitate - *M. mirabilis* (T)	0.22	1.03	9.12×10^{-7}	0.95
digitate - *M. annularis* (L)	0.44	0.65	1.66×10^{-3}	0.47
digitate - *M. annularis* (T)	0.49	0.89	6.02×10^{-6}	0.70
digitate - *A. digitifera* (L)	0.61	0.58	3.63×10^{-3}	0.40
digitate - *A. digitifera* (T)	1.14	0.79	1.86×10^{-5}	0.70
